# Developing mHealth to the Context and Valuation of Injured Patients and Professionals in Hospital Trauma Care: Qualitative and Quantitative Formative Evaluations

**DOI:** 10.2196/35342

**Published:** 2022-06-20

**Authors:** Thymen Houwen, Miel A P Vugts, Koen W W Lansink, Hilco P Theeuwes, Nicky Neequaye, M Susan H Beerekamp, Margot C W Joosen, Mariska A C de Jongh

**Affiliations:** 1 Network Emergency Care Brabant Elisabeth-TweeSteden Ziekenhuis Tilburg Netherlands; 2 Tranzo Scientific Centre for Care and Wellbeing, Tilburg School of Social and Behavioral Sciences, Tilburg University Tilburg Netherlands; 3 Public Health and Primary Care, Leiden University Medical Center Leiden Netherlands; 4 Department of Trauma Surgery Elisabeth-TweeSteden Ziekenhuis Tilburg Netherlands; 5 Department of Trauma Surgery Ziekenhuisgroep Twente Almelo Netherlands

**Keywords:** wounds and injuries, telemedicine, recovery of function, rehabilitation, patient care management, qualitative research, evaluation study, holistic health

## Abstract

**Background:**

Trauma care faces challenges to innovating their services, such as with mobile health (mHealth) app, to improve the quality of care and patients’ health experience. Systematic needs inquiries and collaborations with professional and patient end users are highly recommended to develop and prepare future implementations of such innovations.

**Objective:**

This study aimed to develop a trauma mHealth app for patient information and support in accordance with the Center for eHealth Research and Disease Management road map and describe experiences of unmet information and support needs among injured patients with trauma, barriers to and facilitators of the provision of information and support among trauma care professionals, and drivers of value of an mHealth app in patients with trauma and trauma care professionals.

**Methods:**

Formative evaluations were conducted using quantitative and qualitative methods. Ten semistructured interviews with patients with trauma and a focus group with 4 trauma care professionals were conducted for contextual inquiry and value specification. User requirements and value drivers were applied in prototyping. Furthermore, a complementary quantitative discrete choice experiment (DCE) was conducted with 109 Dutch trauma surgeons, which enabled triangulation on value specification results. In the DCE, preferences were stated for hypothetical mHealth products with various attributes. Panel data from the DCE were analyzed using conditional and mixed logit models.

**Results:**

Patients disclosed a need for more psychosocial support and easy access to more extensive information on their injury, its consequences, and future prospects. Health care professionals designated workload as an essential issue; a digital solution should not require additional time. The conditional logit model of DCE results suggested that access to patient app data through electronic medical record integration (odds ratio [OR] 3.3, 95% CI 2.55-4.34; *P*<.001) or a web viewer (OR 2.3, 95% CI 1.64-3.31; *P*<.001) was considered the most important for an mHealth solution by surgeons, followed by the inclusion of periodic self-measurements (OR 2, 95% CI 1.64-2.46; *P*<.001), the local adjustment of patient information (OR 1.8, 95% CI 1.42-2.33; *P*<.001), local hospital identification (OR 1.7, 95% CI 1.31-2.10; *P*<.001), complication detection (OR 1.5, 95% CI 1.21-1.84; *P*<.001), and the personalization of rehabilitation through artificial intelligence (OR 1.4, 95% CI 1.13-1.62; *P*=.001).

**Conclusions:**

In the context of trauma care, end users have many requirements for an mHealth solution that addresses psychosocial functioning; dependable information; and, possibly, a prediction of how a patient’s recovery trajectory is evolving. A structured development approach provided insights into value drivers and facilitated mHealth prototype enhancement. The findings imply that iterative development should move on from simple and easily implementable mHealth solutions to those that are suitable for broader innovations of care pathways that most—but plausibly not yet all—end users in trauma care will value. This study could inspire the trauma care community.

## Introduction

### Background

Traumatic injuries impose a great physical, psychological, social, and economic burden on victims, relatives, and society. Globally, approximately 1 billion people need health care because of physical injuries [1]. Traumatic brain injuries (55.5 million) and spinal cord injuries (27 million) are the most prevalent types, together causing 17.6 million years of life lived with disability in 2016 [2]. Mostly and increasingly, injured people survive but are confronted with long-term rehabilitation and disabilities in the physical, emotional, and social domains [3,4]. Both in severe injuries and in less severe injuries, patients are at risk for developing symptoms of posttraumatic stress disorder (10%) or depression (7%) or become less productive in their work [3,5,6]. Returning to work is a driver in recovery trajectories, but it is often a lengthy or uncertain endeavor (ie, return to work success rates between 12% and 70% have been found) [7,8].

Health care providers face challenges to innovate their services, such as with mobile health (mHealth) app, to continuously reduce mortality rates as well as to strike the right balance between time and other resource investments and the optimization of patient experiences of health and service quality [9]. For example, *virtual fracture clinics* limit the use of resources in caring for patients with simple and stable fractures while ensuring consistent quality of care [9,10]. This model also appeared to be useful when the COVID-19 pandemic forced (orthopedic) outpatient clinics to limit face-to-face consultations [11]. Another concept aimed to prevent persistent pain symptoms after lower extremity injuries with web-based cognitive behavioral interventions supported by a nurse [12,13]. A preliminary randomized feasibility trial showed less pain intensity in comparison with the provision of an educational pamphlet. As an increasing number of people have internet access, websites, telemedicine, or mobile apps potentially assist in improving patient experiences, accessibility, and cost-effectiveness [14]. Despite the anecdotal success of eHealth and mHealth in trauma care, previous research also showed disappointing adoption, scale-up, spread, and sustenance of communication technologies in health care settings [15]. To prevent common pitfalls, the Center for eHealth Research and Disease Management (CeHRes) road map has been introduced as an evidence-based framework to develop eHealth solutions [16,17]. When technology attributes and features are more complex and stakeholder values are equivocal, the risk of failure increases [15]. Results of one research might not be generalizable across different populations with non–self-selecting injured patients. In this view, failure to address facilitators and barriers in eHealth solutions was associated with nonsuccessful implementation [18]. Therefore, facilitators and barriers should be mapped before developing new eHealth initiatives. By addressing eHealth development through an iterative and collective process of value propositions with all stakeholders, disappointing future impacts can be partially prevented. On the other hand, there is ample literature on the development of eHealth solutions wherein both professionals and patient users collaborate and are subjected to systematic needs inquiries, which is recommended to promote the uptake of eHealth innovations [16].

Therefore, inclusion of both groups could provide new insights in eHealth development.

### Research Aims and Questions

In this study, we aimed to develop an mHealth app serving as a mode to deliver efficacious patient information and support that responds to important requirements of injured patients, health care professionals, and other stakeholders in a Dutch hospital trauma care setting. The CeHRes road map for development [16] was applied in anticipation of future implementation. Herein, we primarily focused on the perspectives of end users: injured patients and trauma care professionals. The objectives were to describe (1) experiences of unmet information and support needs of injured patients to promote their quality of life after hospitalization; (2) actual or expected barriers and facilitators according to trauma care professionals for the provision of information and support (with existing delivery modes or hypothetical innovative propositions) in their work context; and (3) drivers of value of web-based or mHealth apps for both key user groups, that is, the patients with trauma and trauma care professionals. By value, or utility, we mean perceptions (eg, usefulness, relative advantage, and expected outcomes), attitudes, or intentions antecedent to starting (eg, buying or adopting) or continuing app use [15,19,20].

## Methods

### Research and Development Design

**Figure 1:**
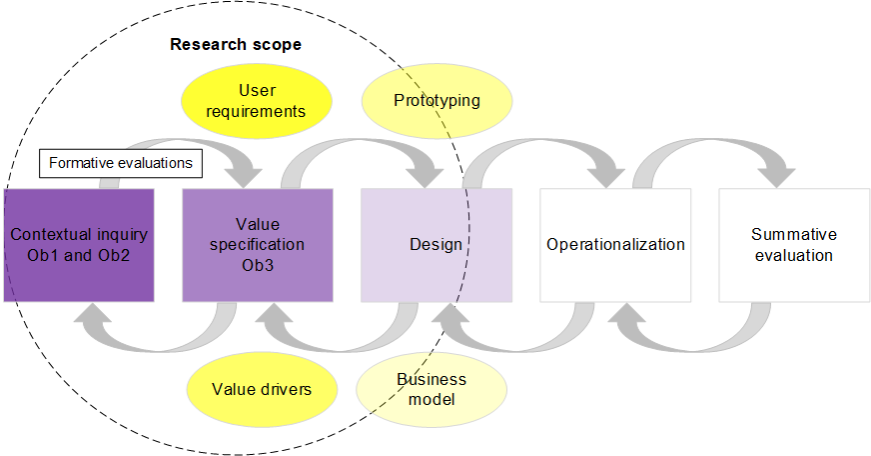
Research scope according to the Center for eHealth Research and Disease Management road map. Adapted from van Gemert-Peijnen et al [17]. The degree of transparency of the shapes (80%-20%) indicates the degree to which each iterative step was completed during the study period of the presented research. Ob: objective.

Development steps were taken according to the CeHRes road map and included “contextual inquiry” (objective 1 and objective 2) and “value specification” (objective 3) [16]. Thus, user requirements and value drivers were established and prototyping was initiated. The scope of the reported steps is shown in Figure 1. Key working principles were “stakeholder participation,” “entanglement of development and implementation,” and “continuous evaluation cycles” [16].

**Figure 2:**
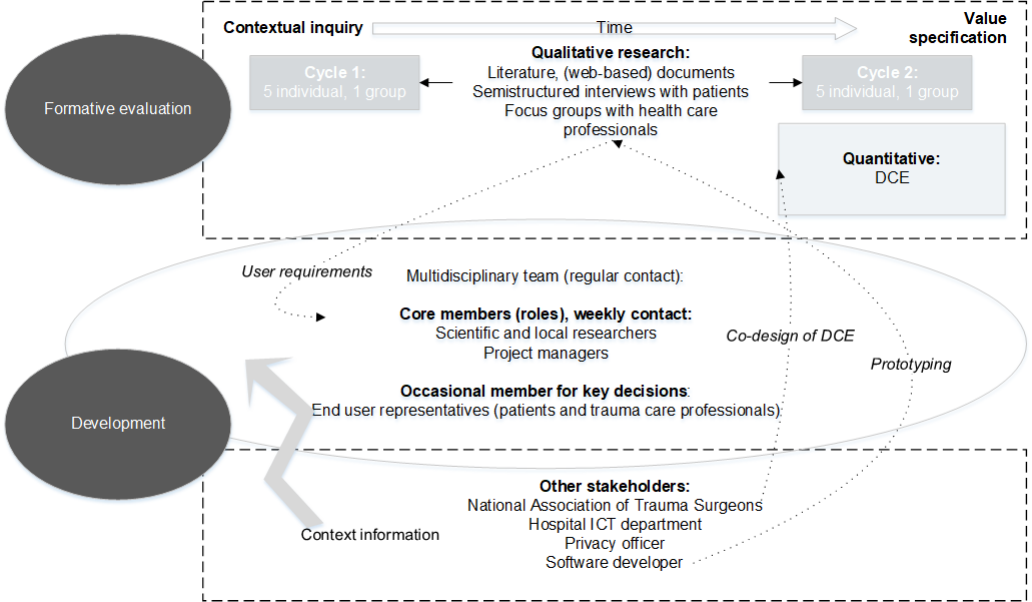
Structure of the development process and formative evaluation design. DCE: discrete choice experiment; ICT: information and communication technology.

Stakeholders involved in the development process were patients with trauma as interview respondents or occasional team members, health care professionals which were mostly trauma surgeons and physiotherapists and in the same roles as patients, hospital information and communication technology (ICT) services, external software developers, the hospital privacy officer, the national association of trauma surgeons, project managers, and researchers. These stakeholders played a role from inside or outside the *multidisciplinary team* (Figure 2). A core multidisciplinary team managed the processes of *steps of the road* in weekly meetings. User representatives occasionally participated when key decisions were made, either by attendance at team meetings with surgeons or separate consultation with patients. Other *external* stakeholders were involved in development in anticipation of future development steps or future implementation.

A formative evaluation using qualitative and quantitative methods was conducted using a (partial) triangulation design [21]. The research methods included interviews with patients with trauma, a focus group with trauma care professionals, and a discrete choice experiment (DCE) among trauma surgeons.

First, qualitative research methods were used to accommodate the action-oriented, explorative, subjective, and in-depth nature of our objectives [22]. Individual patient interviews were completed in 2 cycles. The first interview cycle focused on user requirements and value drivers. These intermediate results were used to inform a phase of mobile app prototype development in collaboration with an external developer. In the second interview cycle, contextual fit and user value were explored using the developed mobile app prototype.

The focus group facilitated interactions between professionals outside routine situations to elicit complex thinking about implementing service changes involving patient information technologies.

Simultaneously, a complementary quantitative DCE with trauma surgeons was conducted to partly triangulate on results of the focus group on the topic of value specification (objective 3) [23]. Participating trauma surgeons stated their preference over hypothetical alternative mHealth products as described by a set of *attributes* and *levels*. For example, one attribute entailed “rehabilitation advice,” which could be *standardized* (level 0) or *personalized* with artificial intelligence (level 1). The DCE simulated trade-offs in deciding between 2 hypothetical mobile patient information apps with varying attributes and price levels. Value hierarchies (priorities) regarding the attributes of patient recovery technology could be inferred based on the chosen alternatives. In addition to an increase in sample size and representativeness (for a national) population of trauma surgeons, quantitative statistical modeling of user value attributions with a DCE provided a way of quantifying preferences and willingness to pay for attributes across decision makers. Ongoing development initiatives and previous qualitative insights obtained by members of an innovation committee from the Dutch Association of Trauma Surgeons (eg, SB) provided a unique window of opportunity for performing a DCE in this target group. There was no such opportunity to also conduct a DCE with patients.

### Setting and Participants

The research took place in the Netherlands, where both surgical and orthopedic trauma care are part of the daily work of trauma surgeons. The qualitative research was conducted in a single level I trauma care center, Elisabeth-TweeSteden Hospital, the designated center for treating severely injured patients within its region. Eligible patients were of working age (18-67 years) and had a traumatic injury from 9 months to 5 years ago. The exclusion criteria were those with (1) a severe traumatic brain injury (ie, Glasgow Coma Score of <8), (2) dementia, or (3) insufficient command of the Dutch language. Focus groups were open to trauma surgeons and paramedics (physiotherapists) who provided direct care to patients from the Elisabeth-TweeSteden Hospital.

The DCE was conducted on a nationwide level, covering level I, II, and III trauma care centers. Dutch trauma surgeons were invited to participate in the DCE.

### Qualitative Data Collection: Interviews With Patients With Trauma

A treating physician (TH, MD, male) selected eligible (former) patients from a trauma registration system and sent research invitations by email. Patient participants (via Microsoft Forms) completed screenings (age, gender, time since hospitalization, and work status) and provided informed consent—for a broader qualitative study. Then, accounting for respondents’ preferred ways of participating and aiming for maximal variation on the screening results, a purposive selection of candidates was sent interview invites, complementary informed consent documents, and screening questions on eHealth literacy and readiness. The participants were provided with a gift voucher of €40 (US $43).

Patient interviews were semistructured and conducted by an experienced researcher (MAPV, PhD, male) via Microsoft Teams. Patients did not establish a relationship with the interviewer before starting the interviews. Each interview lasted 60 minutes and started with an introduction to this study. Next, open questions about experiences of traumatic events, received care and support, (unmet) information and support needs, and the suitability of various modes of delivery were asked. Field notes were taken during the interview. In the second part of each interview, patients were informed of several pre-existing and unevaluated ideas for eHealth or mHealth attributes to deepen their understanding of contextual fit and value considerations [24]. The topic list for the first cycle interviews included short explanations of potential attributes (Multimedia Appendix 1). During the second cycle of semistructured interviews, the list was replaced by a customized prototype containing all current ideas to meet patient user requirements. The prototype was shared on the screen by the interviewer, but patients could, if they wanted to, install and explore the prototype during days before the interview. Multiple screenshots of the prototype are provided in Multimedia Appendix 1. No repeated interviews were conducted.

### Qualitative Data Collection: Focus Groups With Trauma Care Professionals

We planned for 2 to 4 focus group of 90 minutes with 3 to 6 trauma care professionals in each group, which were facilitated by experienced researchers MCWJ (PhD, female) and MAPV via Microsoft Teams. Focus group participant selection targeted trauma health care professionals in the role of trauma surgeons or physiotherapists because of their systematic involvement in aftercare of patients with trauma. The planning of the group meetings adjusted to milestones of the development processes and circumstances related to COVID-19—measures that prevented meeting face-to-face, and time restraints and priorities of hospital staff limiting the opportunity to participate. After introducing the study background and aims, discussions focused on facilitators of and barriers to providing information and support and patient recipient and outcome specifications.

### Qualitative Analysis: Interviews and Focus Groups

After the first interview cycle, one author (MAPV) immersed in the data, relistened the audio files, and made summaries to communicate the user requirements with the development team. After removal of personally identifiable information, verbatim audio transcriptions of all interview material were coded in pairs (TH and MAPV) using Atlas.ti 8 (ATLAS.ti Scientific Software Development GmbH). Relevant fragments of text content about the context and valuation of eHealth propositions were thematically coded using a combination of new labels (open codes) and pre-established codes (ie, sensitizing concepts) [25]. Before coding, a list of potential labels was established and piloted with the help of a third author (MCWJ). This list of potentially applicable codes included concepts from suitable frameworks to describe patients’ health states as context (eg, labels about environmental factors and dimensions of functioning from the International Classification of Functioning and injury types from the International Classification of Diseases) and psychological constructs relating to the *valuation* of technology (perceptions, attitudes, or intentions toward technology use) [19,26]. These frameworks were chosen and discussed explicitly before coding for transparency and consistency. They reflected the complementary backgrounds of the coders in behavioral science (MAPV) and medicine (TH). Using these frameworks as part of the thematic analysis, we identified themes that were clearly defined and embedded in larger frameworks. Open codes were used when important data could not be meaningfully labeled with the listed concepts. In the second coding step, codes were grouped with labels or higher theoretical abstraction, either building on existing frameworks or applying new inducted themes.

### Quantitative Data Collection and Analysis: DCE With Trauma Surgeons

**Figure 3:**
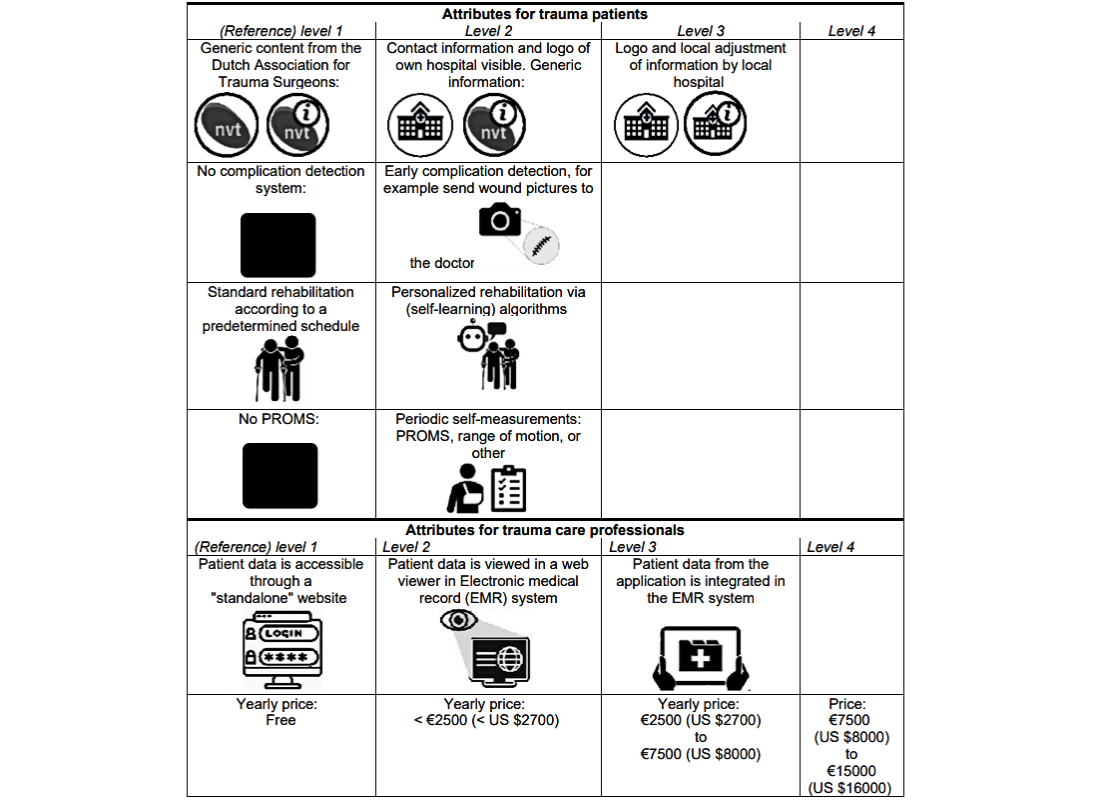
Attributes and levels as in the instrument design. PROM: patient-reported outcome measure.

First, the DCE was conducted according to recommended steps [27]. Additional details for each step are presented in Multimedia Appendix 2 [28]. Hypothetical app attributes and levels were established through a collaboration between researchers and the eHealth working group of the Dutch Association for Trauma Surgeons, represented by MSHB (MD and PhD). Six attributes were established with 2 to 4 different levels (Figure 3), which were refined based on feedback from the eHealth work group about a mock-up of the DCE.

Second, the constructed tasks involved taking repeated and free decisions between 1 out of 2 scenarios of hypothetical mobile patient information and support apps, or to *opt-out* (third option). Herein, opting out may have the relevant meaning of not adopting a newly (jointly) developed solution (*business-as-usual*) [29].

Third, the experimental design was specified in the R language (AlgDesign package) [30]. A total of 11 choice sets were made to adjust to respondents’ limited time to concentrate on the decision tasks; issues with the data of response variability or censoring were considered plausible with a longer test [31].

Fourth, preferences were elicited by informing participants about actual nationwide application development plans and explaining the DCE rationale. Before presenting the choice tasks, attributes and levels were explained as shown in Figure 3. Choice tasks were presented by the question “which application has your preference?” and the answering possibilities “app 1,” “app 2,” or “none of the above apps.”

Fifth, MAPV and MSHB designed the instrument. Hypothetical apps were visually presented as a mobile phone screen with icons of attribute levels shown on 6 planes (Figure 3).

Sixth, the instrument was included in a survey on the priorities of trauma surgeons for patient information resources sent by the Dutch Association of Trauma Surgeons to all members. Similar to a previous DCE with health care professionals under similar circumstances, the board of the association consented to publishing DCE data without obtaining informed consent from individual participants [28]. Complementarily to the choice sets, survey information was used regarding participant characteristics.

Seventh, the panel data from the DCE were analyzed with conditional and mixed logit models using the *gmnl* package in R [32]. Given the use of a convenience sample and a restricted number of choice tasks, sensitivity of the results was checked to various modeling approaches and settings. We have presented figures with results from both the conditional and mixed logit models as either choice has its own advantages and disadvantages. The conditional logit model is a good choice as it requires less data and the results are relatively easy to interpret. Mixed logit models often provide better results as they are more flexible with regard to modeling differences among decision makers but are more data demanding. To illustrate the implications of the modeling results for the valuation of mHealth apps with varying compositions, we calculated the predicted probabilities of selection for 4 different scenarios of attribute (level) combinations as the product of the relevant odds scores (*total odds*) divided by the sum of *total odds* and 1. Other details are presented in Multimedia Appendix 2.

### Ethics Approval

The Tilburg University ethical review board (RP301) approved this qualitative research.

## Results

### Recruitment

#### Patients

A total of 10 individual patient interviews were conducted with 5 male and 5 female patients, with a median age of 58 (range 21-67) years. An invitation was sent to 51 patients. Of the 10 patients, 5 (50%) were interviewed during the first interview cycle and the other 5 (50%) during the second cycle that included a demonstration of the mHealth app. The involved patients had various injuries such as a mild traumatic brain injury or complex or less complex bone fractures of the wrist or ankle. The injuries were because of different traumatic causes (eg, road accident or activity-related injury). All but one were employed, and all were working for the same employer since their injury. The time since the injury ranged from <1 to 3 years. Furthermore, 90% (9/10) of participants regularly used a PC or laptop, 50% (5/10) used a tablet or iPad, and only 10% (1/10) did not use a mobile phone for internet browsing; 80% (8/10) of participants used mobile phones for SMS text messages. Except for 1 patient, all patients previously searched for additional health information and would use the internet for health information. All participants stated that they were sufficiently skillful to find helpful health resources on the internet.

For the second interview cycle, 5 patients evaluated the prototype of a mobile patient app.

#### Trauma Care Professionals

One focus group was conducted with 4 trauma care professionals of which 2 were male trauma surgeons and 2 were physiotherapists (1 male). All participants were working at a level I trauma center.

The respondents to the DCE consisted of 109 surgeons who provided entries for all presented choice tasks. In total, there were 136 survey respondents (136/526, 25.8%), including 134 surgeons who were currently practicing (124/134, 92.5%), in training (9/134, 6.7%), or recently retired (1/134, 0.7%) and 2 researchers and 1 plaster master. Nonresponse (27/136, 19.8% of respondents skipped all tasks) was explained by a problem in visual appearance when the survey was completed via computers with older Microsoft Windows editions that were not used during survey testing. No significant differences were found in the characteristics between respondents who did and did not complete the decision tasks.

Results of contextual inquiry and value specification steps of the CeHRes model are reported separately, but both were addressed during each cycle with a gradual shift in emphasis.

### Contextual Inquiry

The following 7 subthemes concerning information and support needs and associated barriers and facilitators were identified in interviews with patients and focus groups with health care professionals: (1) the need for psychosocial support, (2) information on injuries and consequences of injuries, (3) information exchange between health care providers, (4) experiences of other patients, (5) workload of trauma care professionals, (6) centralized information, and (7) personalized and patient-centered care. Table 1 shows details of the used themes, first-order code groups, second-order code groups, subthemes, and interview quotes.

Patients receiving psychosocial support after having polytraumatic injuries or less complex monotraumatic injuries were positive about the effects on mental health and progression in daily life activities. They experienced psychosocial support as helpful to experiences of anxiety, lack of self-efficacy, or reexperiencing traumas.

**Table 1:** Details of the qualitative part of the study with themes, subthemes, first-order code group examples, second-order code group examples, and interview quotes.

Themes and subthemes			First-order code group examples		Second-order code examples		Interview quote examples
**Contextual inquiry**							
	The need for psychosocial support	1. Patient information and support needs 2. Coping mechanism		1.1. Need for accessibility of biopsychosocial support 1.2. Need for reassurance 1.3. Need for humanistic approach 1.4. Need for information about affective or emotional changes 2.1. Perception of something wrong 2.2. Self-reassurance and cognitive defusion		“A few weeks after the accident, I suddenly started to cry and I could not understand why. A psychologist told me I suffered from psychological trauma. Talking about it and learning the mechanism of psychological trauma supported me in processing this trauma.” [Respondent 5]	
	Information on injuries and consequences of injuries	1. Patient information and support needs 2. Communication between patient and trauma care professional		1.1. Need for information about injury 1.2. Need for information about injury consequences 1.3. Need for information about pharmacological management 2.1 Need for support in signaling and receiving adequate responses to abnormalities or complications		“Additional information in the recovery phase would be of great benefit in comforting me in normal signals and abnormalities, what is actually normal and what is not?” [Respondent 8]	
	Information exchange between health care providers	1. Transfers to or between care settings		1.1. Transfer to other hospital department 1.2. Transfer from general practitioner to emergency department 1.3. Transfer to occupational physician 1.4. Transfer to rehabilitation center		“You need to be attentive as a patient to provide additional information. Important information was most of the times documented, but every now and then, other healthcare providers did simply not see it. This could especially be a problem while having a reduced quality of conscience due to pain medication or illness.”[Respondent 4]	
	Experiences of other patients	1. Coping mechanisms 2. Patient information and support needs 3. Potentially important pre-existing individual differences between patients		1.1. Dealing with fear of consequences 2.1. Need for social support 2.2. Need for information about affective or emotional changes 3.1. Expected individual difference in social support seeking 3.2. Expected individual difference in psychological vulnerability		“I had no need for a support group with peers, but reading about experiences of others supported me in realistic prospects and expectation management.” [Respondent 2]	
	Workload of trauma care professionals	1. Physician time restriction or work load 2. Development and implementation factors 3. Target outcome for app		1.1. Availability specialist 1.2. Asynchrony in available time between patient and physician 1.3. Change in share of routine vs nonroutine tasks 1.4. Recognition of physicians time scarcity 2.1. Reducing trauma care provider burden required for adoption 3.1. Reducing trauma care provider burden required for adoption 3.2. Reducing clinical visits		“Physiotherapists sometimes contact me in the weekends by using the communication app X (a previously introduced application) to discuss certain patients. Of course, it is my own decision to answer questions outside normal working hours, but there are already so many ways in which our tasks are being extended, that too accessible communication by patients with the doctor would be undesirable.”[Trauma surgeon 1]	
	Centralized information	1. Development and implementation factors		1.1. Preference that patients use only one app 1.2. Implementation requirement: back office or response to process patient input		“Several applications are being offered to patients, but as a patient, I would expect that all information is summarized in one tool and all communication is possible within this same tool.” [Physiotherapist 1]	
	Personalized and patient-centered care	1. Evaluations of health services 2. Patient information and support needs		1.1. Attitude toward existing services 1.2. Limited specified information 2.1 Need to personalize patient information and support to varying or unknown actual needs		“I hope, it is possible to build an application which can be self-learning to improve our standardized care.” [Trauma surgeon 2]	
**Value specification**							
	Suggestions for improvement of psychosocial and mental health	1. App attribute ideas		1.1. Hypothetical app attribute: (intelligent) monitoring and benchmarking of progress in QoL^a^ and functioning (in rehabilitation phase) 1.2. Proposed app attribute: collect, model and deploy patient health data (ie, signal and responding to red flags) 1.3. Proposed app attribute: facilitated access to psychosocial help 1.4. Proposed app attribute: open field to tell your story about the event		“People should take matters into their own hands, you can assist them in monitoring psychosocial health, but they must draw their own conclusions about normality and abnormality to search for additional help on time.” [Respondent 3]	
	Information on injuries and consequences of injuries	1. Valuation of app attribute ideas 2. App attribute ideas 3. Target outcome for app		1.1. Attitude toward (hypothetical) technology 1.2. Perceived ease of use of (hypothetical) technology 2.1. Hypothetical app attribute: Information about common symptoms that are no reason of concern 2.2. Proposed app attribute: frequently asked questions 2.3. Hypothetical app attribute: informing about treatment procedures 3.1. Need for perspective 3.2. Recovery		“I have seen many people with functional illiteracy. Terms and language were supposed to be absolutely clear, but were not common for quite some people. That is the moment when people drop out.” [Respondent 7]	
	Suggestions for videos and visuals vs textual information	1. App attribute ideas		1.1. Need for guiding information or videos or photos 1.2. Proposed app attribute: possibility for sound input		“You could even add more images and graphics. A lot of people lose focus when too much text appears.” [Respondent 10]	
	Using surveys to detect a deviating course	1. Communication between patient and trauma care professional 2. App attribute ideas		1.1. Advantage for both patient and physician 2.1. Hypothetical app attribute: Stimulate active interaction with app to personalize content 2.2. Hypothetical app attribute: wound picture 2.3. Proposed app attribute: collecting questions or observations		“In the beginning, I was too focused on the recovery and rehabilitation of my ankle; the implications on my future life were secondary.” [Respondent 9]	
	Work-related information	1. Activities and participation (ICF^b^ d8) 2. App attribute ideas		1.1. (Return to) work and employment (ICF: d840-d859) 1.2. Acquiring, keeping and terminating a job (ICF: d845) 2.1 Hypothetical app attribute: prompt communication with employer		“A general advice and a prospective view on return to work would be of great benefit. I do know I have to contact my employer, but when can I start working again?” [Respondent 6]	

^a^QoL: quality of life.

^b^ICF: International Classification of Functioning.

Some patients did not receive any psychosocial support, although they required psychosocial assistance. Patients did not receive support as health care professionals failed to offer it, patients did not realize the need to ask for additional support, or patients did not know where to find psychosocial support. Therefore, patients suggested standardizing the possibility of talking about emotional consequences with a social worker, spiritual caregiver, or psychologist during hospital admission. In addition, the patients suggested providing information on where to find additional psychosocial support after hospital admission.

Extensive information on injuries and injury consequences could reduce the uncertainty of physical recovery and improve the ability to cope with limitations in daily life. Patients searched for specific information that could be used as a resource for additional information or to reread previously informed information. Participants did not demand for complicated and detailed medical information, but they appreciated receiving basic information about the injury, treatment or treatments, complication risks, how to use painkillers, prospective on rehabilitation, and a useful prospect about the process (steps or duration) of rehabilitation. Three patients suggested using animations or short videos to discuss these topics.

Some people also missed the future prospects for returning to work. Although participants were generally satisfied with the guidance from their occupational physicians, they sometimes missed the context and information from their in-hospital health care provider. In particular, information about how injuries implied work limitations, the time span to return to work, and whether one could reasonably expect to become able to return to the *normal* working situation.

Several participants felt that communication between health care professionals from different disciplines was limited. Patients believed that most of the information was exchanged via electronic patient files and letters. The absence of face-to-face communication between health care providers may cause a loss of information. One patient with a traumatic brain injury felt that he always needed to be alert to notice mistakes during hospital admission.

Exchanging experiences with other patients often recurred during the interviews. Patients looking for leads to improve their own physical and mental health mentioned the need for like-minded experiences. Rehabilitation experiences and duration were the most commonly mentioned factors. The main goal was to obtain an impression of the illness or trauma and its subsequent consequences. Some patients had no interest in directly sharing their experiences with other patients with similar conditions. Only a few patients searched for a support group to exchange experiences, tips, and tricks, and these participants experienced benefits from a support group.

Trauma care professionals experienced several barriers and facilitators in daily trauma care. Workload was an important theme mentioned as it can act as a barrier or facilitator in introducing new web-based information tools or apps. Potentially, eHealth could relieve health care providers by supplying additional information to patients. However, the use of a mobile app or web-based application should never result in extra workload for trauma care professionals. For example, the communication capabilities of eHealth solutions should not overload professionals by shifting more work to doctors and bypass triage nurses.

Health care professionals emphasize the usefulness of an app for measurement and triage for patients potentially sustaining complications after an operation or injury. Hence, standardized but unnecessary visits might be reduced, and a shift can be made between patients who need to be seen after 2 weeks and those who can be seen after a longer period. Furthermore, 2 health care providers suggested an artificial intelligence function in which questions were automatically directed to the responsible caregiver of the specialist. This is used to balance the workload.

Different health care professionals have highlighted the importance of a centralized patient information and reporting platforms. Currently, many different platforms and tools are available, but patients and health care professionals emphasize that a single tool would be beneficial. For example, reports or information on physical functioning from patients and physiotherapists or questionnaires could be available to the trauma care professional. This information could subsequently be used in following advice and treatment.

One health care provider summarized the use of eHealth as the ultimate goal to learn from patient outcomes to provide better targeted therapies and to adjust treatment where necessary.

### Value Specification: Interviews With Patients and Professionals

On the basis of the first contextual inquiries, an existing mHealth app (patient journey) was modified and shared with patient participants during the second round interviews to facilitate value specification. These interviews revealed perceptions, attitudes, and intentions to use an eHealth application. The following five themes were identified: (1) suggestions for the improvement of psychosocial and mental health, (2) information on injuries and consequences of injuries, (3) suggestions for videos and visuals versus textual information, (4) using surveys to detect a deviating course*,* and (5) work-related information. Table 1 presents details of the qualitative part of the study.

In addition, 60% (3/5) of participants responded positively to the proportion of self-monitoring psychosocial health outcomes. Patients and professionals agreed that data about psychosocial health, obtained by an mHealth app, could especially be used by professionals to facilitate appropriate referrals to colleagues (ie, psychologists). Furthermore, 2 patients suggested to include free text blocks in addition to structured surveys to express psychosocial difficulties. One patient suggested the use of charts for illustrating scores as a result of the questionnaires (ie, numerical pain rating score). This could indicate improvement or deterioration that requires action (receive additional advice of guidance or contact health care professionals).

Participants experienced the information in the prototype as extensive and detailed. A “read more” button for additional information was perceived as needed by 3 patients to prevent information from being too extensive. Some concerns focused on the level of education for which an app should apply. All participants agreed with the appropriateness of the current information.

Most patients missed the appropriate information on painkillers during their own recovery phase. Therefore, patients experienced information on the frequency and use of painkillers as useful. The prototype contained information on paracetamol only, but patients also requested information on additional posttraumatic pain medications such as nonsteroidal anti-inflammatory drugs and morphine.

The prototype included textual information and instruction videos, and 80% (4/5) of participants experienced this as advantageous and suggested using more videos to instruct on mobilization, exercises, or consequences of injuries. Graphical content would be easier to understand.

The prototype suggested some solutions for earlier recognition of complications and complication management. Patients especially appreciated clear information about *red flags* and normal symptoms after treatment. Questionnaires for complication follow-up or as detectors for a deviating course or new complications were also suggested. Generic questionnaires (eg, patient-reported outcome measures or the Patient-Reported Outcomes Measurement Information System) could be used for monitoring of pain, physical function, and social or mental health. All patients stated that short surveys could be helpful in detecting problems. One patient suggested a diary function including a timeline for monitoring complaints.

In the prototype, expectations and advice on rehabilitation and recovery were divided into time spans after injury. For example, in terms of physical function, weight bearing after an operation differs between 1 and 6 weeks. All participants were positive about this feature. One patient stated that information on expectations should not be stated too early during the rehabilitation phase.

The prototype contained general advice to early contact the employer and occupational physician. Participants were generally satisfied to early contact the occupational physician and the remark that returning to work could take weeks to months, but some also questioned this general character, as working situations can differ between patients and personal advice in an app could be difficult to state. Furthermore, some stated that guidance in returning to work should be in the hands of the occupational physician, but the information on where to find additional guidance could be implemented in an app.

### Value Specification: DCE With Trauma Surgeons

Results of the DCE enabled us to triangulate on parts of the interview outcomes. Multimedia Appendix 2 provides a complete overview of the statistical results on the recruitment of trauma surgeons and from the predictive modeling of the surgeons’ discrete choices.

**Figure 4:**
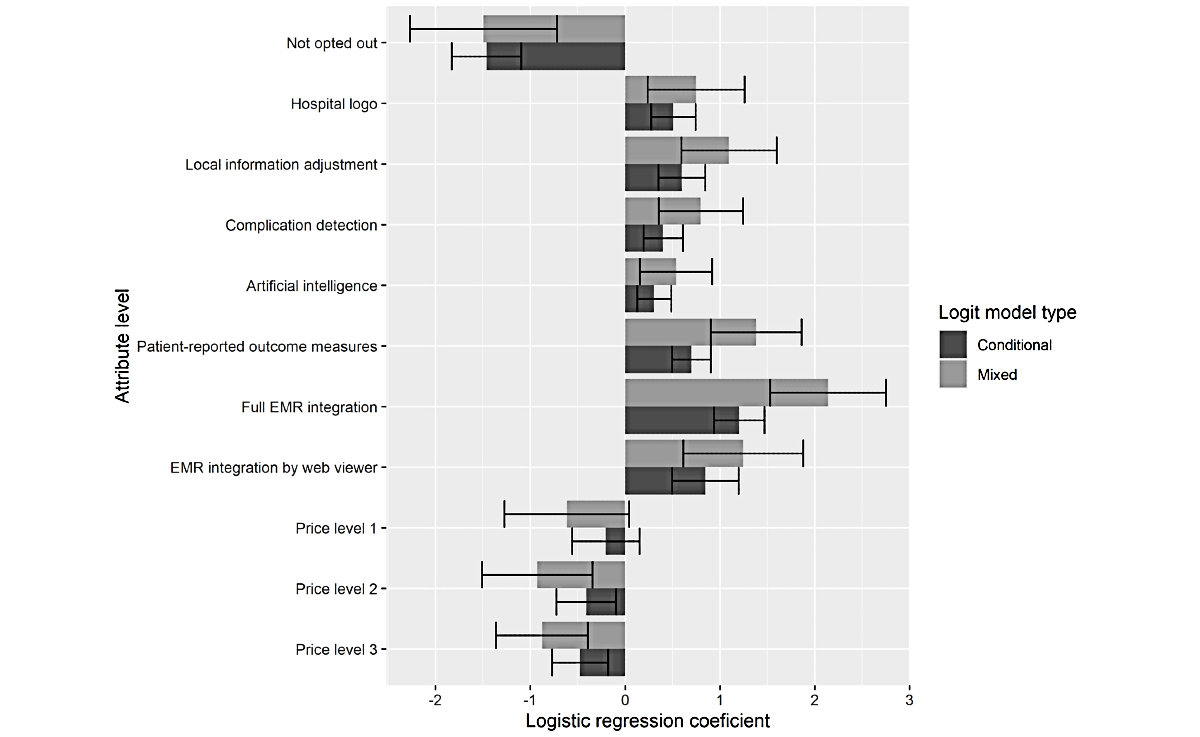
Weight estimates with 95% CIs for model 1 attribute levels. EMR: electronic medical record.

**Figure 5:**
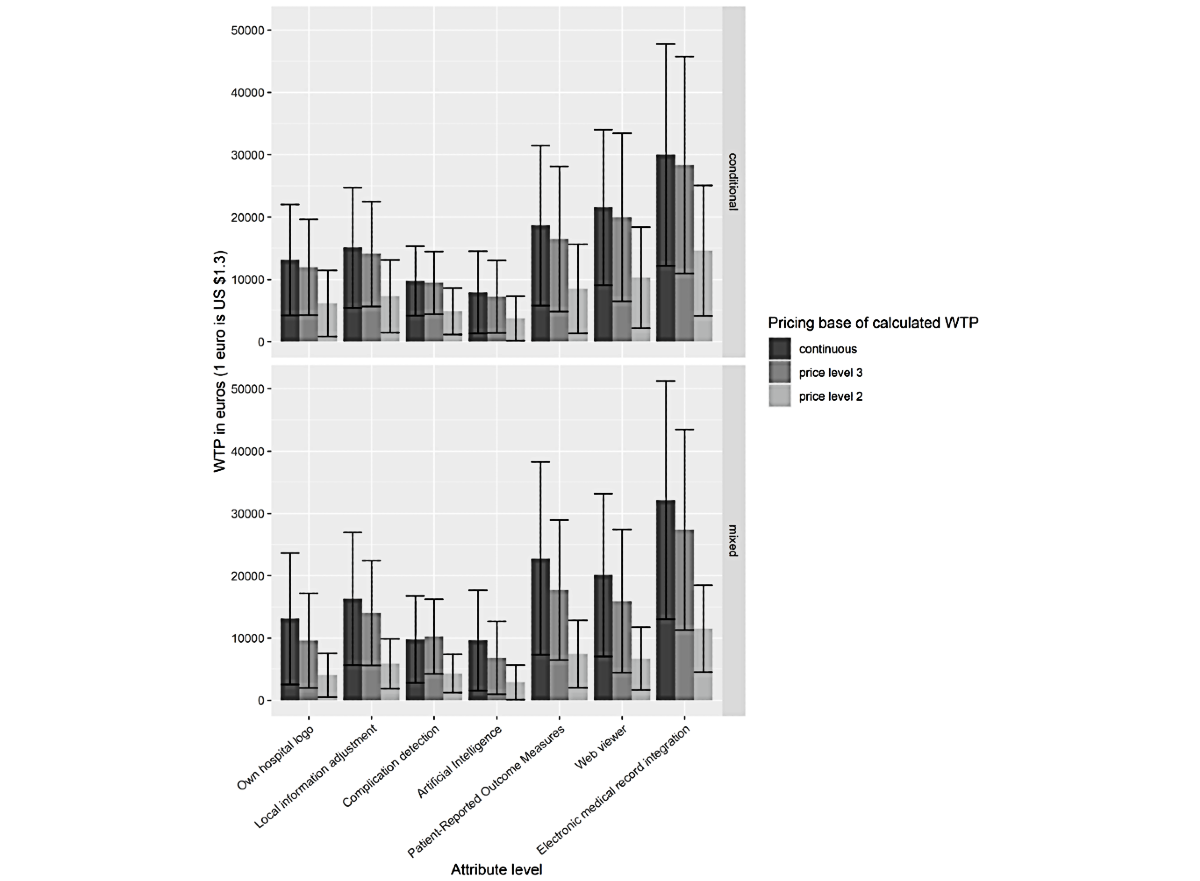
Willingness-to-pay estimates with 95% CIs and their sensitivity to different methodological choices. WTP: willing to pay.

Consistently across the analyses of respondents’ discrete choices, patient app data access through electronic medical record integration (conditional logit odds ratio [OR] 3.33, 95% CI 2.55-4.34; *P*<.001) was weighted the highest. This was followed by the inclusion of a web viewer (the second level of the patient data access attribute; OR 2.33, 95% CI 1.64-3.31; *P*<.001); periodic self-measurements (OR 2.01, 95% CI 1.64-2.46; *P*<.001); the local adjustment of patient information (OR 1.82, 95% CI 1.42-2.33; *P*<.001); local hospital identification (OR 1.66, 95% CI 1.31-2.10; *P*<.001); complication detection (OR 1.49, 95% CI 1.21-1.84; *P*<.001); and, lastly, the personalization of rehabilitation through artificial intelligence (OR 1.36, 95% CI 1.13-1.62; *P*=.001; Figure 4). In contrast, the estimates were negative (and OR<1) for price levels below €2500 (US $2700; OR 0.82, 95% CI 0.57-1.16; *P*=.26), between €2500 (US $2700) and €7500 (US $8000; OR 0.66, 95% CI 0.48-0.91; *P*=.01), and between €7500 (US $8000) and €15,000 (US $16,000; OR 0.62, 95% CI 0.46-0.83; *P*=.002). The implications of the results for the estimate of willingness to pay, and its sensitivity to methodological choices, are visualized in Figure 5. This reveals wide CIs, such that the lower bound for willingness to pay was almost 0 for artificial intelligence personalization and the upper bound for electronic medical record integration was >€40,000 (US $42,761; annual). In contrast to what is expected with rational decisions, the difference between the weights for the price levels of €2500 (US $2700) to €7500 (US $8000) and €7500 (US $8000) to €15,000 (US $16,000) was relatively small compared with the absolute monetary value difference, which complicates the interpretation of willingness-to-pay results.

Furthermore, the findings suggested significant variation in preference weights across respondents. Specifically, the improvement of model fit between mixed logit model 1 (McFadden pseudo *R*^2^=0.27; *good*) and conditional logit model 1 (pseudo *R*^2^=0.08; not *good*) was substantial. Furthermore, models (ie, model 2) with fixed interaction effects between preference weights and respondent characteristics improved the choice predictions. The odds of selecting an app were relatively higher in surgeons with less than 10 years of work experience (*β*=.78, SE 0.14, Exp[*β*]=2.20; *P*<.001) and in those who rated the need for collective app development (very) high (*β*=.79, SE 0.14, Exp[*β*]=2.19; *P*<.001).

These weights can be used to estimate the likelihood of selecting an app under various scenarios. For example, the odds of selecting a hypothetical app with only *basic level* attributes were 1 over 5, corresponding to a probability of 0.18. Among health professionals with >10 years of experience and without a high need for collective development, the estimated probability of selecting such a basic was 8.6%. Table 2 provides estimated probabilities of app selection under 4 different scenarios.

**Table 2:** Selection probability estimates for 4 difference scenarios of app compositions.

Scenario	Attribute levels	Estimated probability of selection, %	
		Conditional logit	Mixed logit
Basic app for free	Generic logos and information No communication options for complication detection Standard rehabilitation plan No patient health monitoring No patient medical record integration of patient data access No costs	18	19
Best possible attributes, highest price	Hospital logo and local information adjustment Complication detection Personalized rehabilitation plan through artificial intelligence Patient health monitoring Full medical record integration €7500 (US $8000) to €15,000 (US $16,000 per year	78	97
Best possible attributes except EMR^a^ integration workaround	As above with full medical record integration replaced with web viewer	71	94
The two most preferred attribute levels and price level 2	Generic logos and information No communication options for complication detection Standard rehabilitation plan Patient health monitoring Full medical record integration €2500 (US $2700) to €7500 (US $8000) per year	49	76

^a^EMR: electronic medical record.

## Discussion

### Principal Findings

This study took the fundamental steps of contextual inquiry and value specification in the development of mHealth to provide injured patients with the information and support they need to recover and improve their quality of life after treatment in a (Dutch) trauma care center. We coordinated the development process among various stakeholders (eg, aligning with other initiatives with overlapping scope, addressing privacy concerns, and managing processes of integration within hospitals’ existing ICT services) while maintaining a focus on end user perspectives to increase chances of future patient uptake, health care professional adoption, implementation, and scale-up across (Dutch) trauma care settings. Various formative evaluations enabled us to better understand (1) injured patients’ (unmet) information and support needs, (2) barriers and facilitators in the current work of health care professionals to provide information and support provision (either with or without eHealth solutions available to them), and (3) drivers of value of an mHealth app for key user groups: patients with trauma and trauma professionals. Emerging insights informed prototyping: Selection, adjustment, and testing with an *off-the-shelf* solution with potential contextual fit. Following these steps (of the CeHRes road map) showed that eventually both trauma care professionals and patients share the need for highly accessible and valid information on how a patient’s trajectory of recovery is evolving. An mHealth app including information exchange between the patient and the trauma care professional would ideally lead to a prediction model that facilitates personalized recovery trajectories.

Formative evaluation revealed important lessons. First, qualitative examinations of the (unmet) information and support needs revealed patients’ need for psychosocial support and easy access to more extensive information on their injury, its consequences, and their future prospects, including return to work. Additional support and information could reduce experiences of uncertainty during physical recovery and improve the ability to cope with limitations in daily life. Second, inquiry of barriers and facilitators in the current working context of health care professionals suggested that workload is a crucial issue with regard to eHealth solutions. mHealth solutions can either act as barriers (shifting more work to doctors) or facilitators (work relief) in introducing new tools or eHealth or mHealth apps. Third, this finding explains why our DCE among trauma surgeons identified data access through electronic medical records as the most preferred attribute. In addition, albeit to a lesser extent, trauma surgeons appeared to value hospital level information adjustment and identification and personalization of rehabilitation through artificial intelligence. According to the DCE results, few trauma surgeons may be inclined to start using an mHealth app that meets none of such requirements. This seemed to be even truer the longer a surgeon is in the profession. Both the patients and professionals responded positively to using mHealth for monitoring by administering short surveys on complications, pain, physical function, and social or mental health; and receive valid feedback or prepare for inpatient consultation visits.

### Comparison With Prior Work

The findings of our study add to the existing literature aimed at overcoming barriers to the successful development and implementation of eHealth initiatives that followed the CeHRes road map [16,20,33-40]. Previous studies have addressed a wide range of eHealth initiatives such as the development of a web-based intervention for depression [37], a computerized clinical decision support system for patients with type 2 diabetes [36], an mHealth intervention for patients with prediabetes [33], and a digital training tool to support oncologists in the skill of information provision [40]. However, only one earlier study addressed a more traumatic oriented topic [38]. Although the CeHRes road map has been used in this study, numerous other frameworks are available to develop eHealth or mHealth initiatives [41]. For example, the agile science approach that includes an iterative process and focuses on flexible concepts to develop and test eHealth prototypes [42], or the persuasive system design model that focuses on influencing behavior in a positive manner [43]. Other examples include intervention mapping or the Accelerated Creation-to-Sustainment model [41]. Instead of selecting a single right development model for addressing key concerns, it is preferable to select and combine research methods to address the needs, demands, and values of end users and important stakeholders [41].

Our formative evaluations provided a relatively rare qualitative perspective on factors and domains of health-related quality of life with regard to patients’ desire of well-delivered information and support during an episode of hospital trauma care. Both psychological (ie, coping, anxiety, self-efficacy, and a future prospective on the return to work) and social needs (ie, family support or a contact person in the hospital) were highlighted as essential for quality of life and progression in restoring daily life activities. Other studies have shown the influence of various consecutive (transfers between) contexts of care and support, including hospital trauma care centers, rehabilitation services [44], primary care [45], and social environments such as family [46] and work [8].

Previous studies have indicated that professionals in trauma care setting could play an important role in managing factors related to quality of life. Patient motivation, self-awareness (eg, cognitive impairment), self-efficacy (eg, managing pain or returning to work), social interaction, (work) goal setting (eg, changing occupation, following education), and eHealth or mHealth solutions could support in efficient patient guidance [7,8,47,48].

However, our qualitative look at these possibilities also highlighted that different contexts belonging to individual patients complicate the development of an eHealth app that is both personally meaningful and scalable.

### Strengths and Limitations

To our knowledge, this study is the first to use the CeHRes road map for the development of a mobile delivery mode for information and support to improve experiences and multidimensional health outcomes of injured patients from trauma care settings. Using the CeHRes roadmap helped prevent common development pitfalls such as *supply drive*, *reinventing the wheel*, or a *not invented here* mindset [16,41]. During the development process, different stakeholders were involved that represented both the *demand side* (ie, patients and professionals) and the *supply side* of a *value proposition*, which is pivotal for adoption, scale-up, and maintenance of eHealth initiatives [15]. Although only patients and professionals were formally included in the study, hospital ICT services, a hospital privacy officer, and an external software developer were involved in the brainstorm sessions and prototype development. Methods used in formative evaluation during development were diverse and applied in a creative manner.

Furthermore, there were specific strengths and limitations to these formative evaluations. Our purposive sample included patients from a Dutch level I trauma center with variable injury types and complexities. This heterogeneity of patients promoted the generalizability of our findings across trauma populations.

Between the short development iterations, we embedded a quantitative method within a broader qualitative approach to triangulate on value specification from the perspective of (Dutch) trauma surgeons. Conversely, the use of qualitative data from the interviews and the focus group supported the creation of *attributes* and *levels* and compensated for the simplifying assumptions that DCEs make about complex *real-world* value attributions.

Nonetheless, several limitations to our qualitative and quantitative methods co-occurred with the challenges of aligning formative evaluation steps with those of app development and of COVID-19 restrictions. First, the foreclosure to recruit and interview patients face-to-face hindered the selection and representation of views of patients with lower levels of *eHealth literacy*. Moreover, the strategy to select a maximally heterogeneous sample of 10 patients for semistructured interviews was a choice of convenience: working toward data saturation or seeking for sampling heterogeneity in more traumatic injury dimensions were considered impractical given our goal to timely support app development with short cycles of formative evaluation. For example, a large subpopulation of patients with traumatic brain injury was represented by one patient who was also a source of insight into the uniqueness (eg, hospital boundary crossing) of the specific recovery trajectory and the associated differential needs; the needs for information related to hospital transfers and cognitive rehabilitation are minor themes within the analysis that are significant for the individual patient. Second, owing to restricted time schedules, the number of *formal* focus group meetings performed (ie, one) and the heterogeneity of roles in hospital trauma care represented in that group (trauma surgeons and physiotherapists) were smaller than desired. Third, qualitative methods mostly focused on patient perspectives, whereas quantitative methods only addressed value specification from the perspective of trauma surgeons. Thus, the principle of data triangulation was applied to part of our research scope. Full compensation for the sample size limitations of the qualitative methods would require an extended DCE sample including patients and other types of health care professionals. Consequently, it should be taken into account that our sampling strategy may have limited the generalizability of our findings regarding contextual barriers and facilitators and valued app attributes across different patient subpopulations and professional roles in trauma care. Finally, we did not perform quality assurances in the form of member checking or return interview transcripts to interview participants for possible corrections.

### Implications and Recommendations

This study shows the diversity in the needs and opinions of patients and trauma care professionals regarding (digital) information provision in the current trauma care. Our findings clearly reflect the common value among patients with trauma and trauma care professionals to pursue more efficient and better-informed trauma care. A more efficient exchange of specific, valid, and standardized injury-related information and more convenient monitoring of physical, mental, and social health can be achieved by using an mHealth app. A trauma care professional who receives more information about a patient’s physical, mental, and social health can better adapt his or her treatment to the individual patient. Moreover, secondary use of patient data can develop prediction models for a broad range of relevant health aspects. These models can subsequently be used to further improve shared decision-making by better-informed patients and doctors. However, this study also reminds us that such an eHealth initiative requires the broad support of patients, trauma care professionals, and other stakeholders.

The insights we obtained for the development of an eHealth prototype that fits the needs of essential end users were already used and can now be used by others to improve eHealth or mHealth apps for patients with trauma. For further development of a broadly supported eHealth app for trauma (after) care, a structural development process, for example, by means of continuing the CeHRes road map, is recommended [16].

Other authors or researchers could use this paper as inspiration for future eHealth projects in related fields of research. Herein, we recommend developers to be open to continuing iterations on each step, despite the fact that the process will move at a slow pace. The context cannot be inquired *completely* in a single cycle with regard to all-important matters to all the stakeholders. For example, future implementation of coordinated after care supported by an mHealth solution may depend on how the sustained provision of such a service is paid for, or on legal issues regarding information exchange between patients and health care professionals of different facilities. Future studies could focus on the expansion of participants on the *supply side* and *demand side* in which, for example, outpatient nurses and clinical ward nurses could be involved in qualitative assessments. Moreover, it is strongly recommended that additional development based on contextual inquiry and, when suitable, prototype testing should emphasize on less computer or internet literate patients. When insurmountable contextual barriers are met, it is better to know them and to make the solution simpler to promote future implementation. In this regard, virtual fracture clinics provide an example to build upon.

### Conclusions

This study reveals that most end users in trauma care do not just need any app or mHealth solutions. Patients, particularly those with complex injuries, require psychosocial support and easy access to more extensive information about their injury and possible journey toward recovery. Both patients with trauma and trauma care professionals strive for dependable information and, possibly, a prediction on how a patient’s trajectory of recovery is evolving. Using the CeHRes road map, we were able to develop a mobile delivery mode based on the needs of both patients and trauma care professionals with basic computer skills. The formative evaluation process made it possible to iteratively adapt and improve the current prototype in an efficient way. This study could potentially serve as a starting point for future development of eHealth or mHealth initiatives within the trauma care community.

## Appendix

Multimedia Appendix 1 Topic list—semistructured interview with patients.

Multimedia Appendix 2 Additional details of methods of the discrete choice experiment.

## Abbreviations

CeHRes: Center for eHealth Research and Disease Management
DCE: discrete choice experiment
ICT: information and communication technology
mHealth: mobile health
OR: odds ratio
