# Supplementary material for: Developing mHealth to the Context and Valuation of Injured Patients and Professionals in Hospital Trauma Care: Qualitative and Quantitative Formative Evaluations
Source: JMIR Hum Factors. 2022 Jun 20;9(2):e35342. doi: 10.2196/35342 (PMC9254041; doi:10.2196/35342)

Multimedia Appendix 1. Topic list—semistructured interview with patients.

**Introduction**

Topics – *example questions*

Experiences

- Traumatic event
- Processes of recovery: acute treatment, rehabilitation, adjustment, stabilization (‘new normal’)
- Retaining, losing and / or returning to work
- Support and guidance of the Elizabeth Tweesteden Hospital (ETZ)

*Could you tell me about the event that led to your injury: what happened?*

*How did you fare afterwards; compared to the situation before the event?*

*How has your situation developed over time, until now?*

*How did your recovery with regard to working related activities developed over time? (That means, short before, short after, a few months and years after the accident)*

*During what kind of activities or in what kind of activities was additional support, guidance or assistance desired after the medical treatment?*

- *From the Elisabeth Tweesteden Hospital*
- *From other Healthcare professionals*
- *During work*
- *During ‘self-aid’*

Needs for support and guidance

*What is your opinion on the need of support and guidance?*

*Did the provided support matched with the needs at that certain moment?*

*What kind of support was abundant?*

*What kind of support was missing?*

Experiences with:

- The way health services (inside and outside the ETZ) offer guidance and support for a broad spectrum of health issues.

*Can you tell something about the practical implementation of additional support of different involved healthcare providers?*

*Expenses in time and funding?*

*How did different healthcare providers collaborated?*

Needs for digital support in healthcare problems

- What kind of devices or tools did you used as support for health/recovery/return to work, which were not offered from healthcare providers?
- Digital support

*During your recovery, have you ever used for example websites, applications or a smartwatch to find additional information, advices, support in recovery or support from other healthcare professionals?*

*If yes, what kind of digital support? Were tools or devices available from the ETZ (e.g. ‘mijn ETZ’)? What motivated you to start using certain tools? What is your opinion on used tools?*

*If not, what was holding you back to use additional tools or devices?*

**‘Prototypes’**

**Cycle 1:**

Depending on previous answers, current available websites and applications for this certain patient population can be outlined. Important features of other digital support will then be discussed as background information.

*It is fairly difficult to ask an opinion on something that has not been developed yet. That is why I would like to explain the possibilities of implementing new features in an application especially focused on trauma patients. Examples of possible features are now being piloted within other hospitals or units within the ETZ. Involved stakeholders do have high expectations of these features, but usage is limited and implementation is difficult. Healthcare providers do not often refer patients to available tools. Would you like to evaluate the available features to discuss the most essential and beneficial features?*

‘Treatment-guidance or Behandelwijzer in Dutch’: *This application is stepwise showing general information which is specified by diagnosis. Important phases and steps with regard to treatment, recovery and support are outlined. It assesses how patients generally cope with the injury and recovery, shows essential information at specific time-points and advices for example on how to cope with pain.*

‘Distant physiotherapeutic support’: *support in recovery exercises: ‘personal’ training schedule with videos, adjusted to your current situation and registration of progression and performed exercises.*

‘Decisional aid’: *A website with information about treatment possibilities. This has already been developed for patients with fractures and limitations in daily life.*

‘Diaries’: *It can sometimes be hard to give an accurate and reliable view on how things fare outside the hospital setting. It is important though for both your own interpretation and the interpretation of the doctor to outline difficulties and problems. An extensive questionnaire at one moment in time or discussing your difficulties with a healthcare provider does have some limitations. Instead of or in addition to these standard ways of evaluation, it could be possible to provide patients with a ‘beep’ in which they ask to answer a short questionnaire or question at random moment. This in order to monitor how often pain occurs or to evaluate the patients’ mood and to see at what moments for example this pain or feelings occur. Both you and the doctor receive a summary of the information, for example in a line graph.*

‘Distant physical monitoring’: *Physiological measurements and information can be monitored by using a smartwatch or pedometer. Just like more subjective information, physiological measurements could be processed in the electronic patient file so that healthcare professionals can monitor their patients from a distance.*

‘Functionalities for social contact’: *Get into contact with peers or receive information from peers and inform the immediate surroundings.*

*All functionalities in an application can be more and more personalized if they are using information about your own personal situation. However, the exchange of information and the linking of multidimensional information has some important privacy aspects with for example an external login page. What is your opinion about these privacy aspects?*

**Cycle 2: Patient journey application (screenshots are on the next page)**

The application is at this moment only available for patients with a fracture of the ankle.

*Imagine that you suffered a fracture of the ankle instead (of own previous injury)*

*Would you like to download the application and view the content? In that case, I will just leave you with the application and grab some coffee. If you need some assistance, just let me know.*

*What is your first impression?*

Explore every chapter/part in the application if needed:

*Can you imagine that this kind of information could be useful? How? When?*

Evaluation (Final questions):

- *What are the best/most beneficial elements of this application? Why?*
- *Are there any downsides in this application? Why?*
- *Do you have any suggestions for improvements or new elements that could be implemented in the application?*
- *Does this application fit in your current situation? If yes/no, why?*
- *How could this application be modified to fit better into your own situation?*

**Screenshots Patient Journey App**


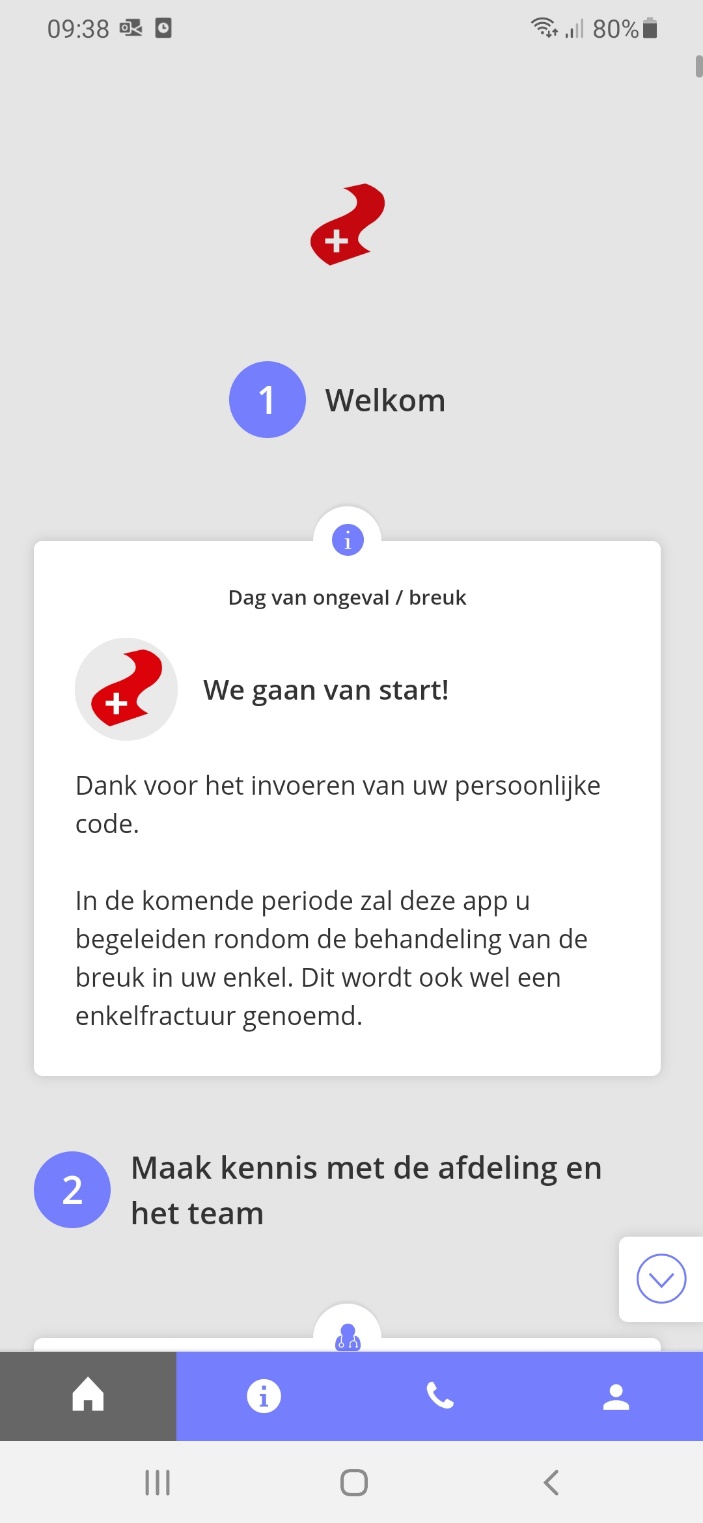

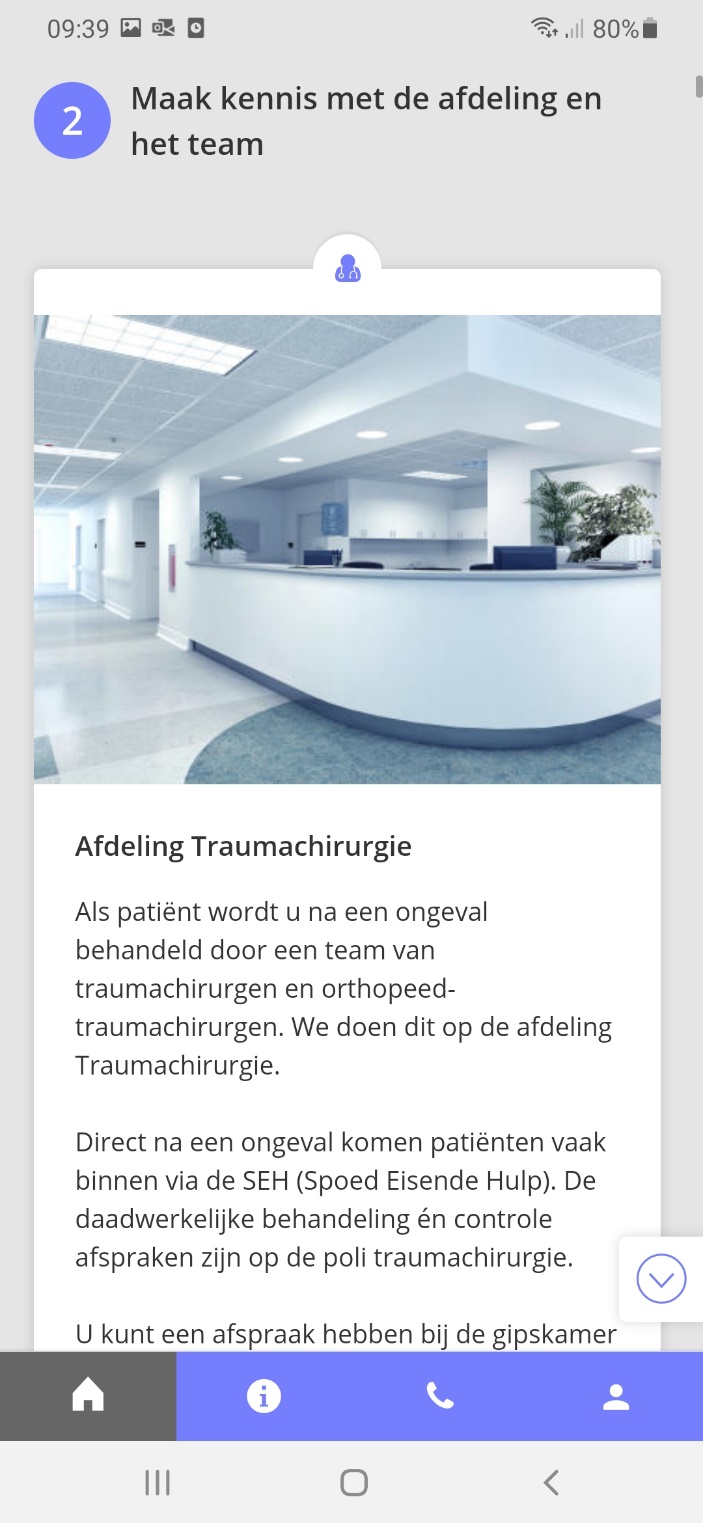


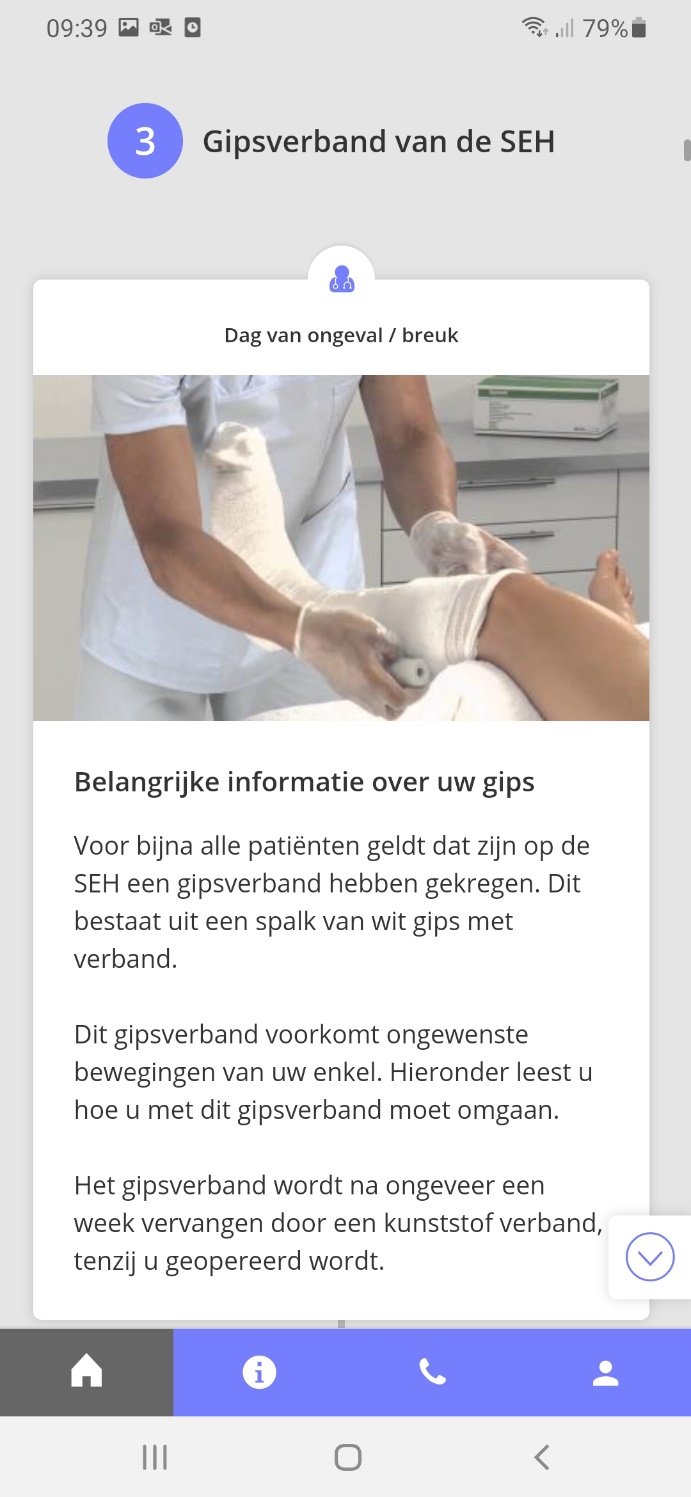

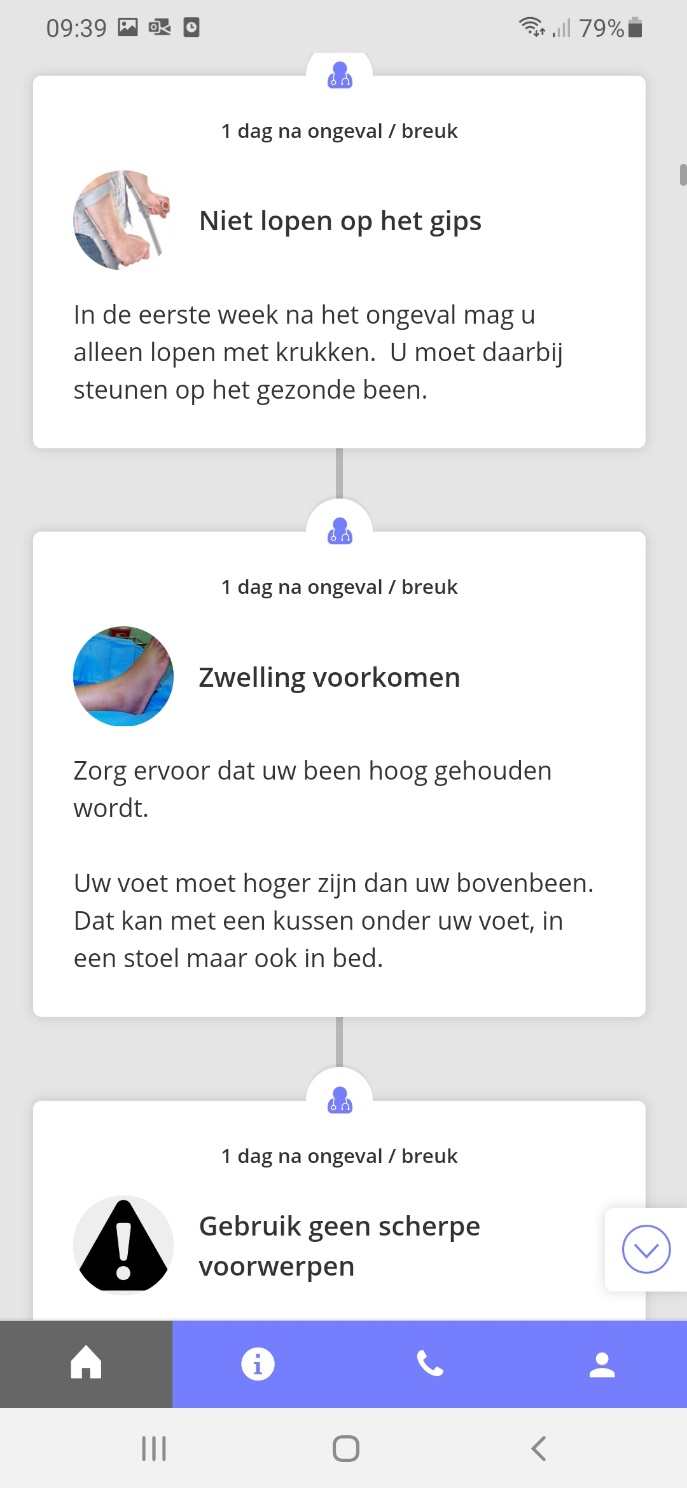


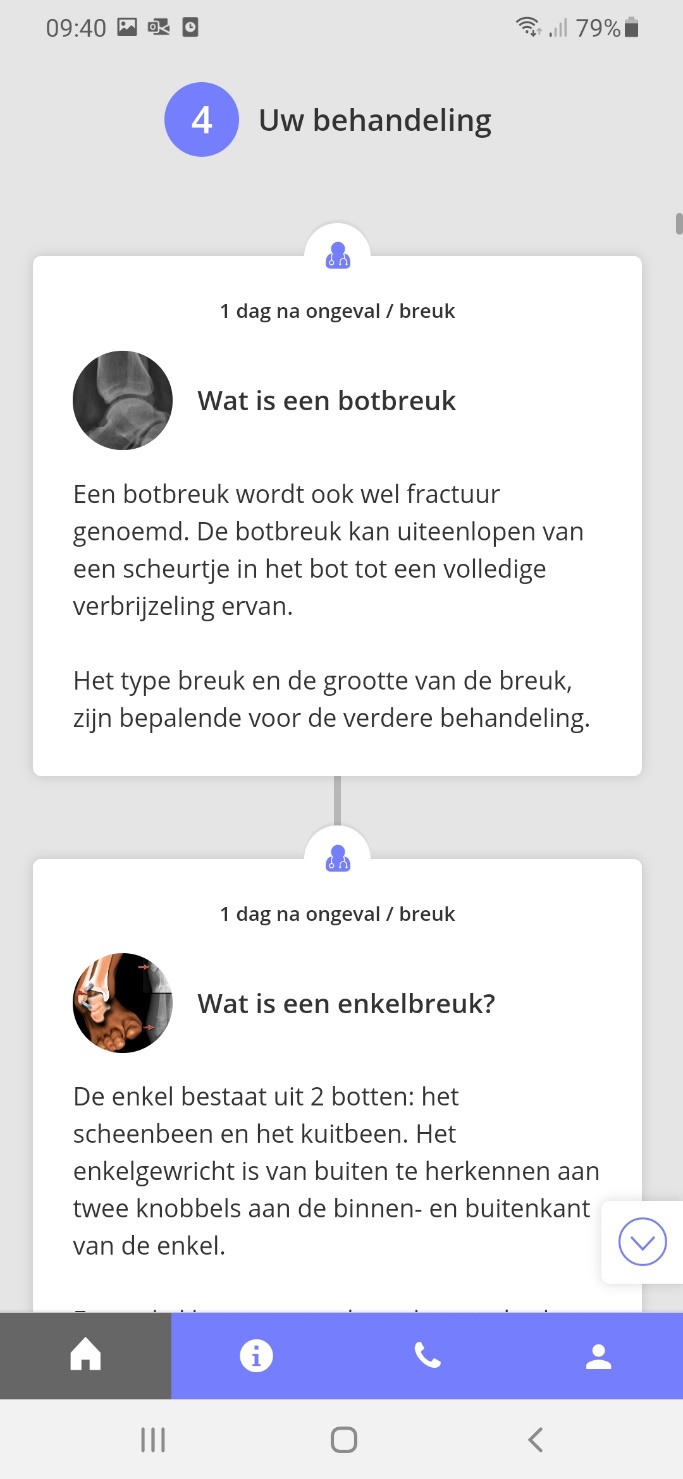

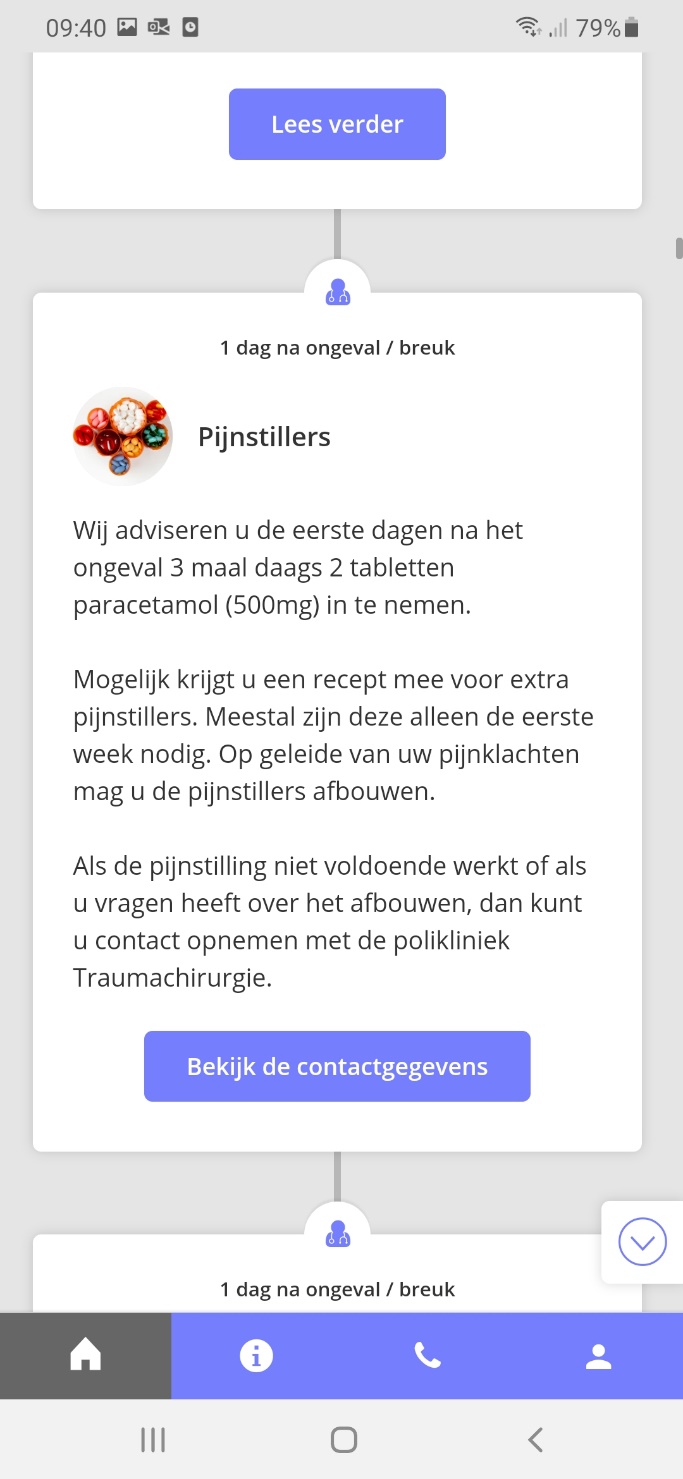


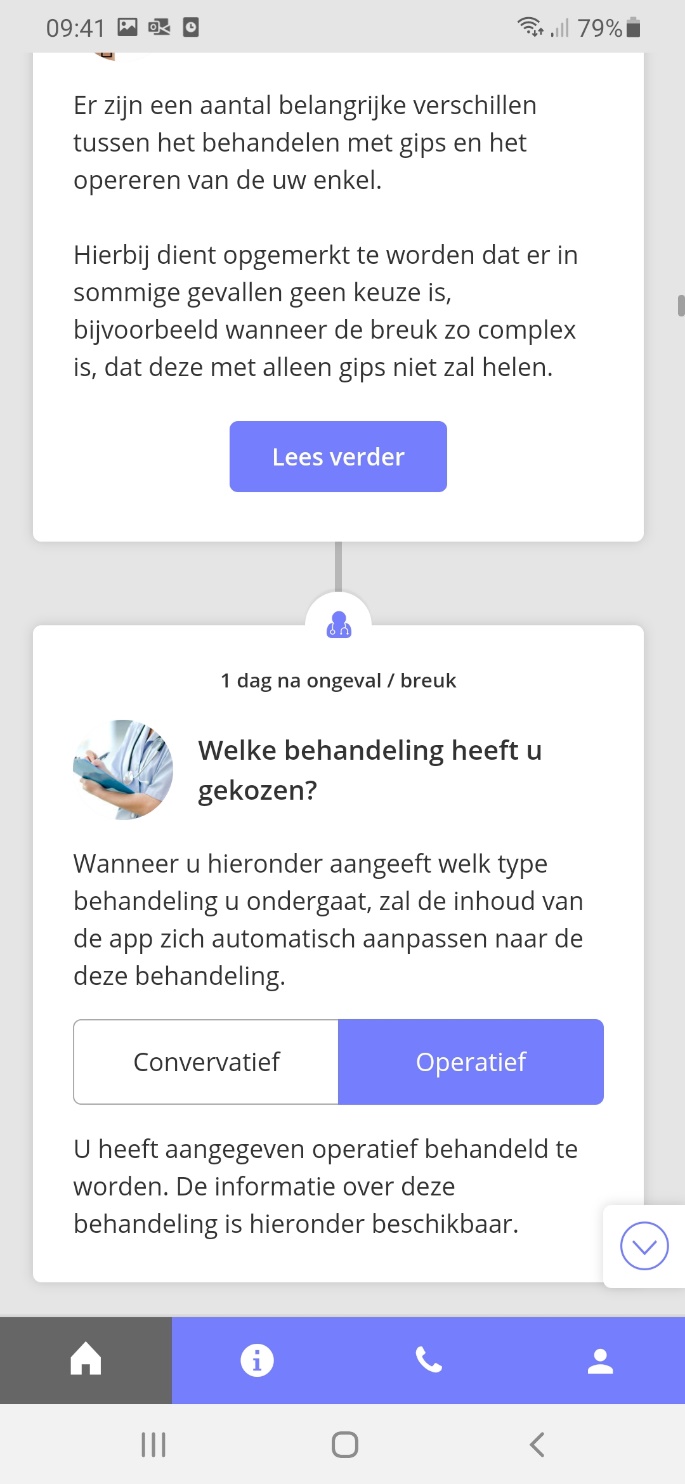

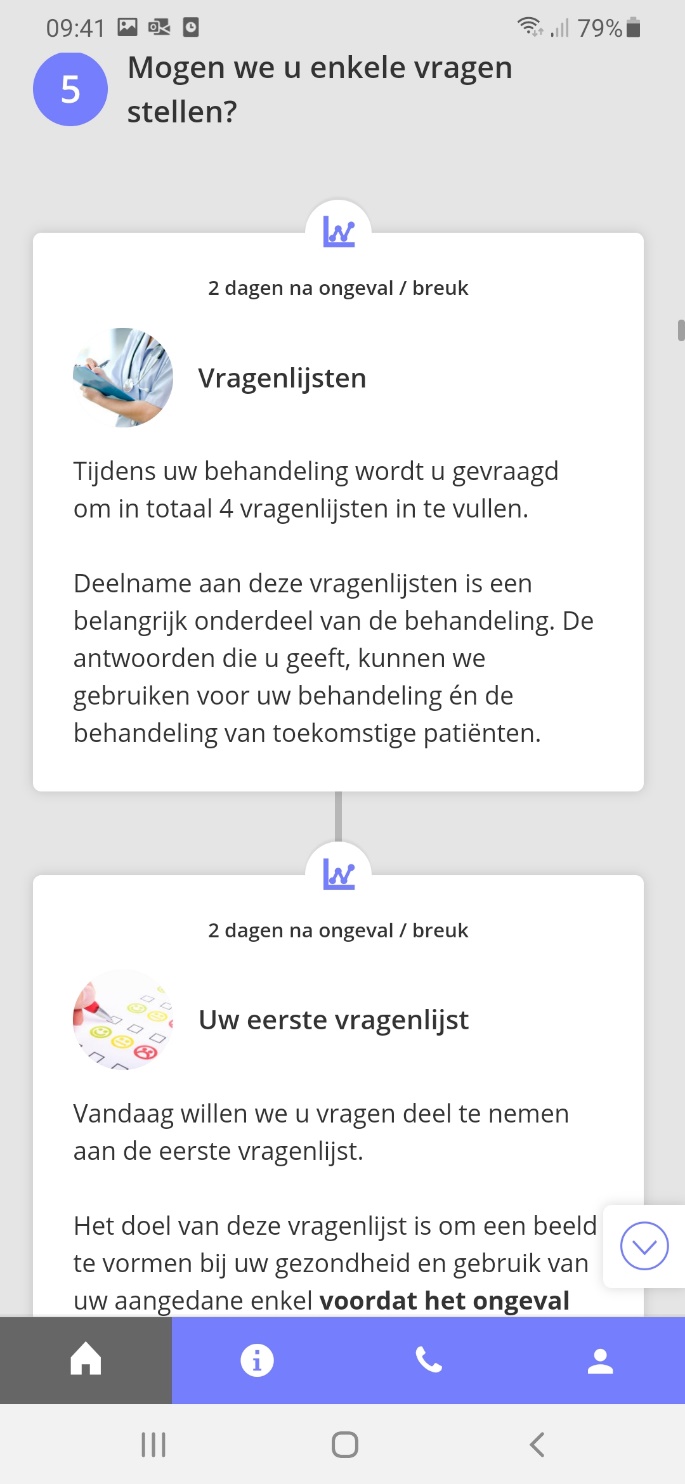


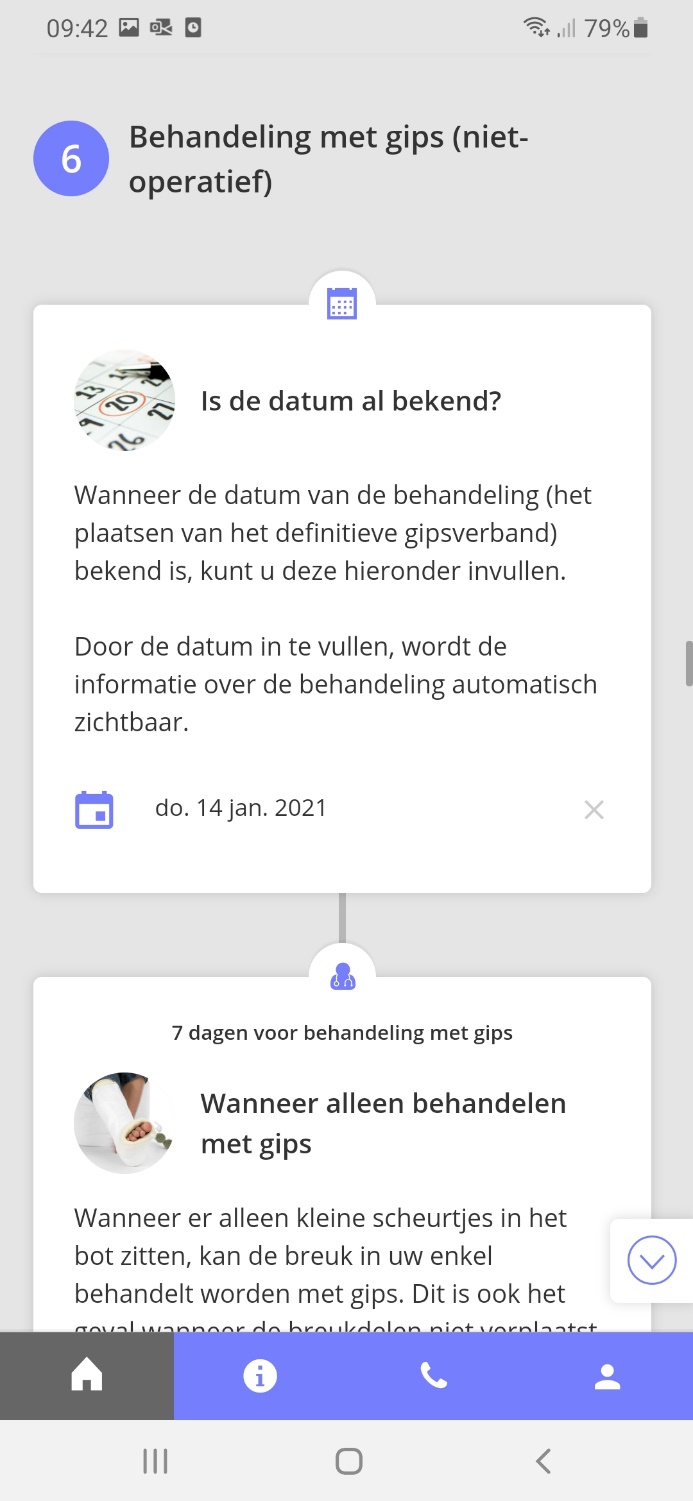

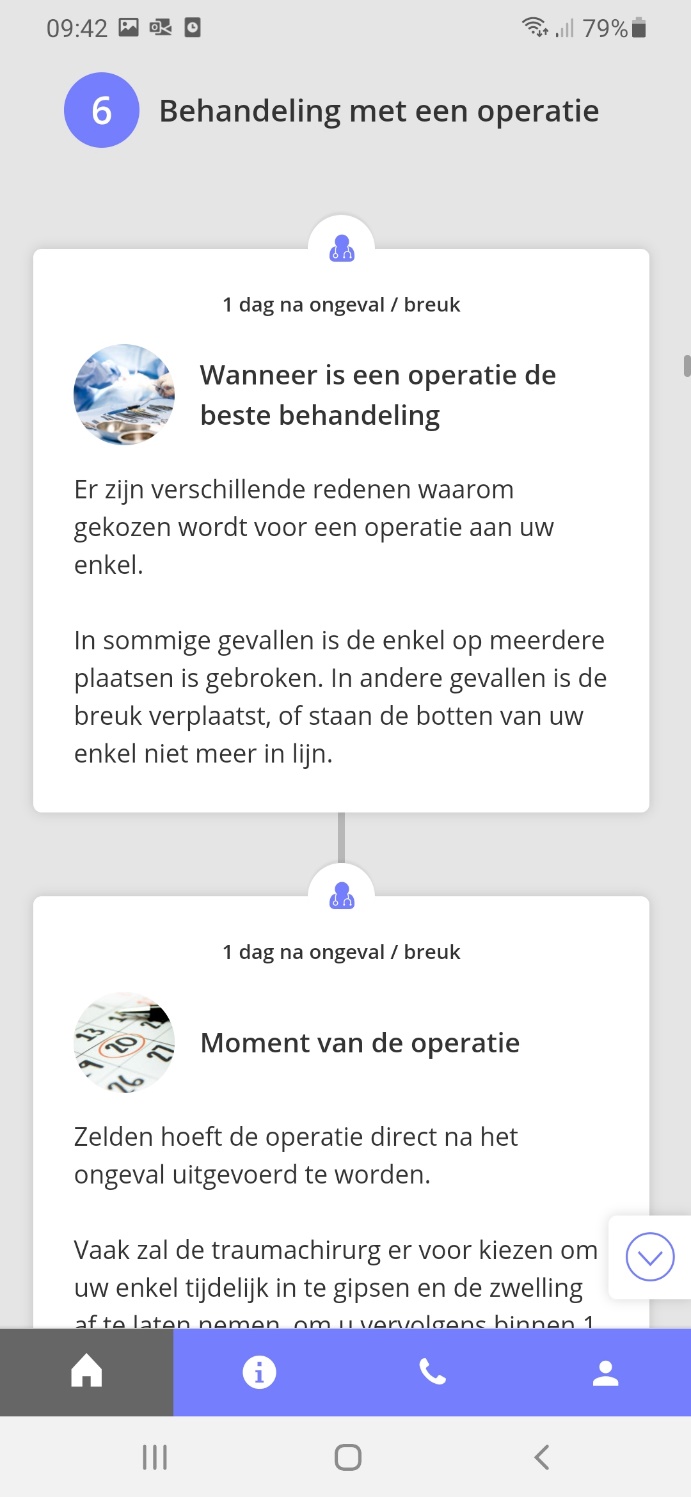


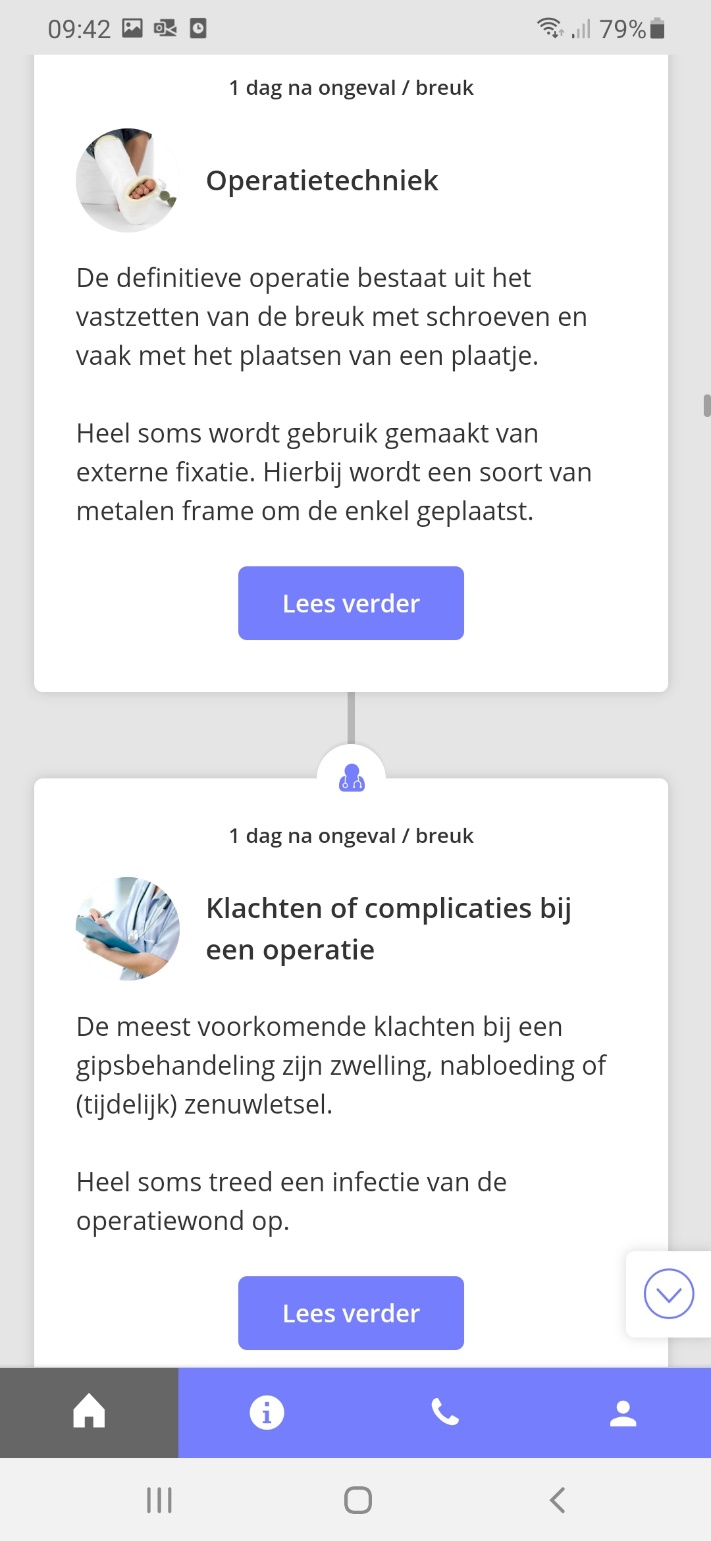

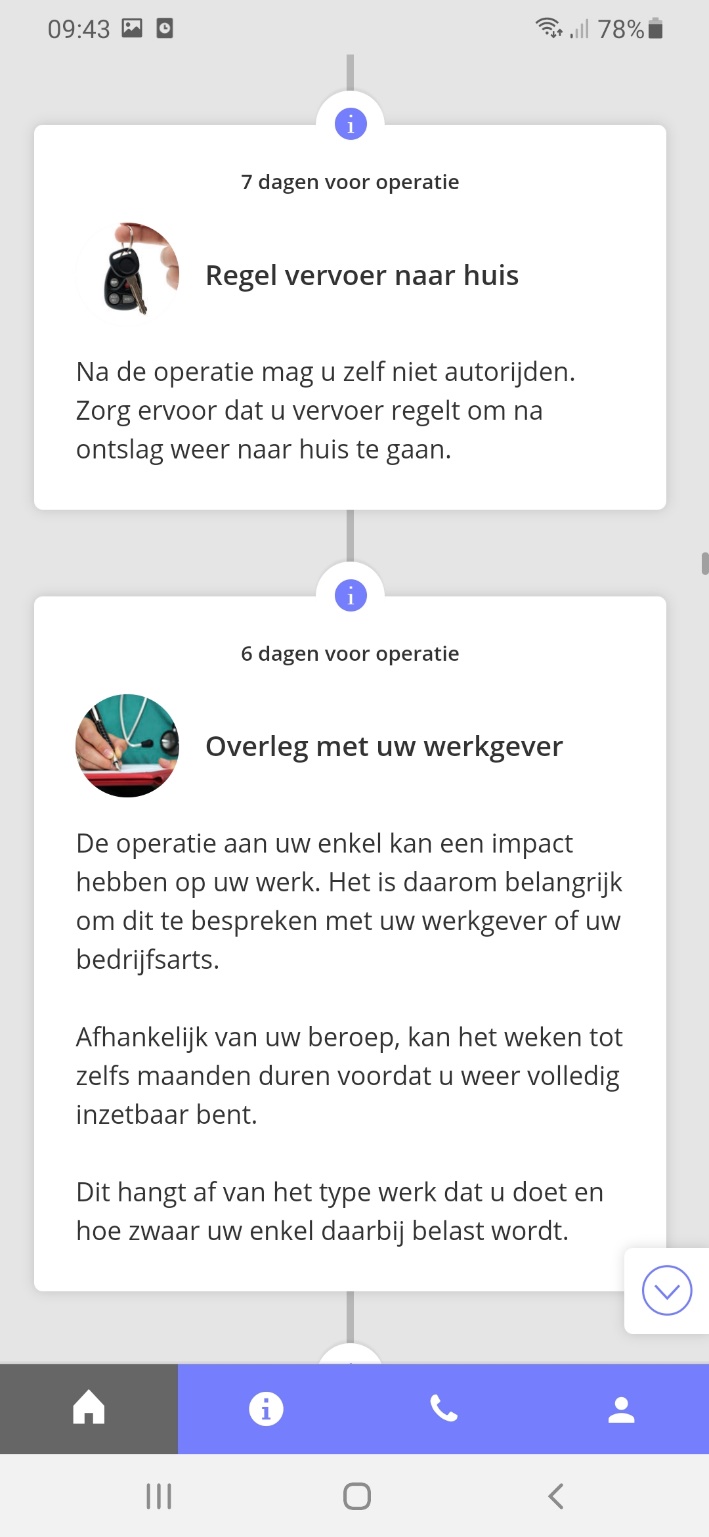


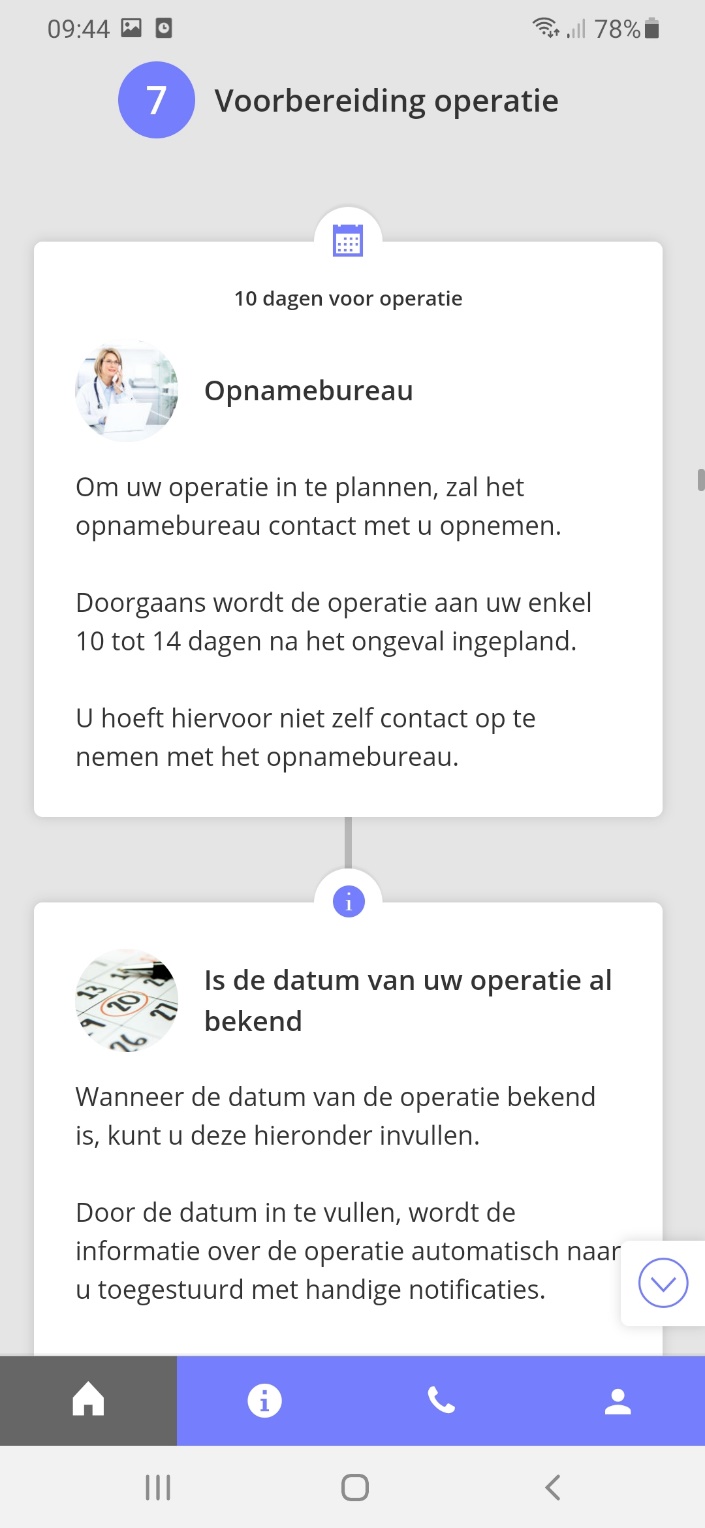

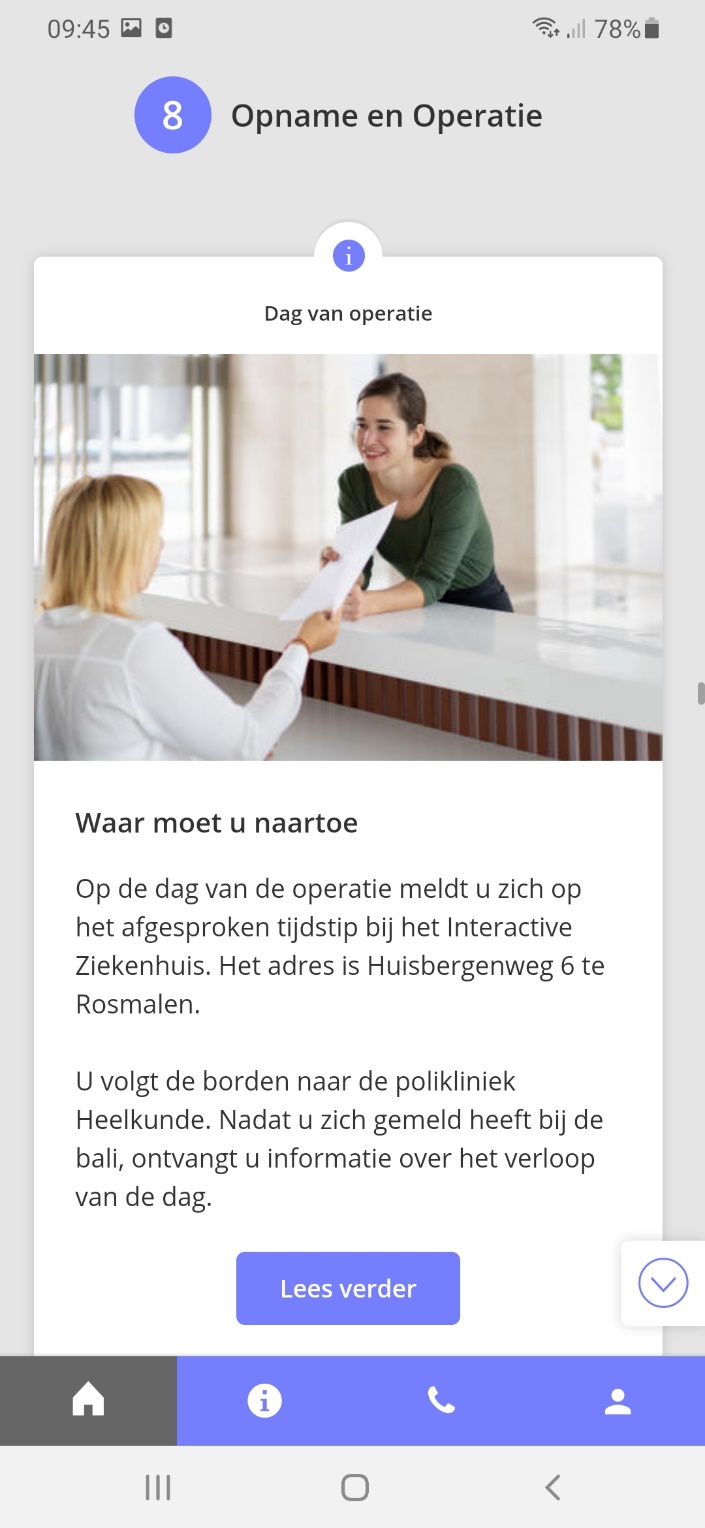


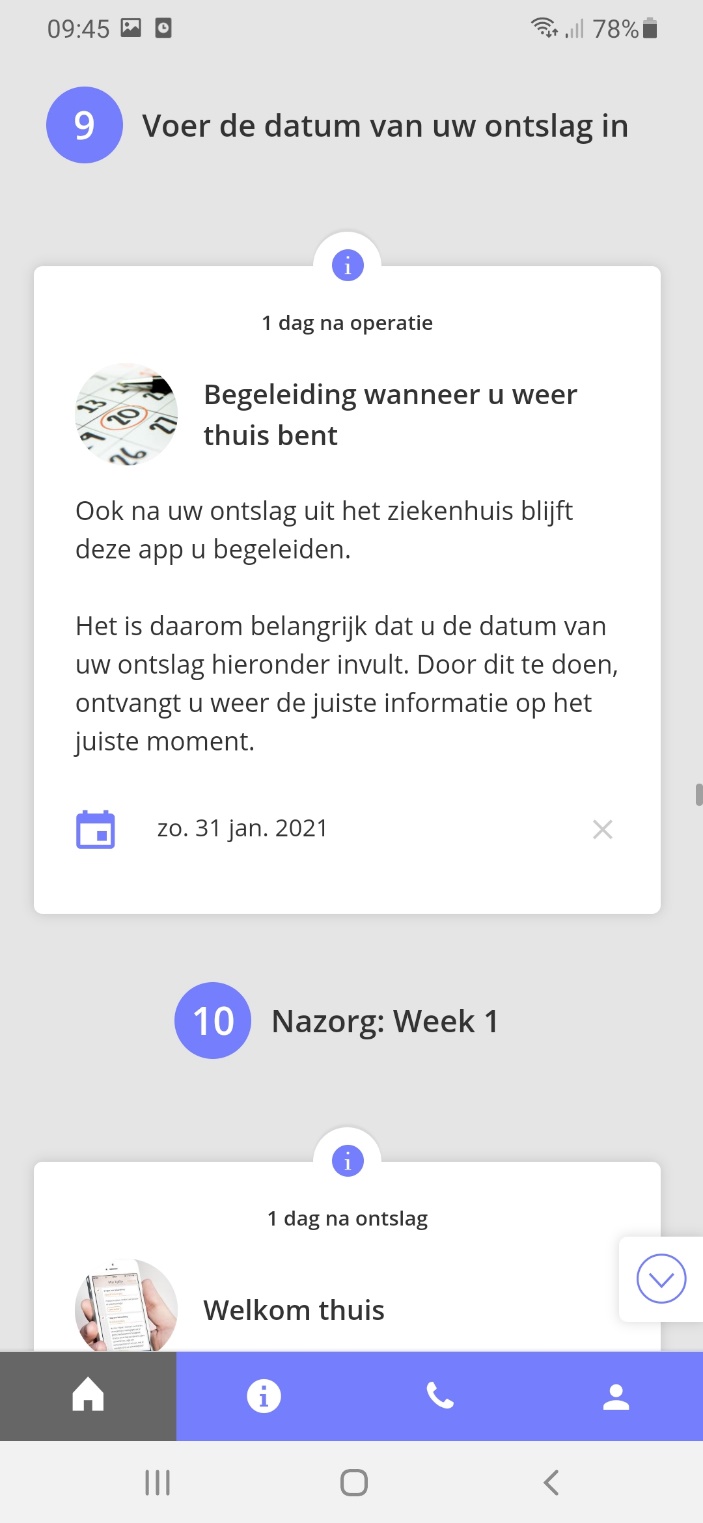

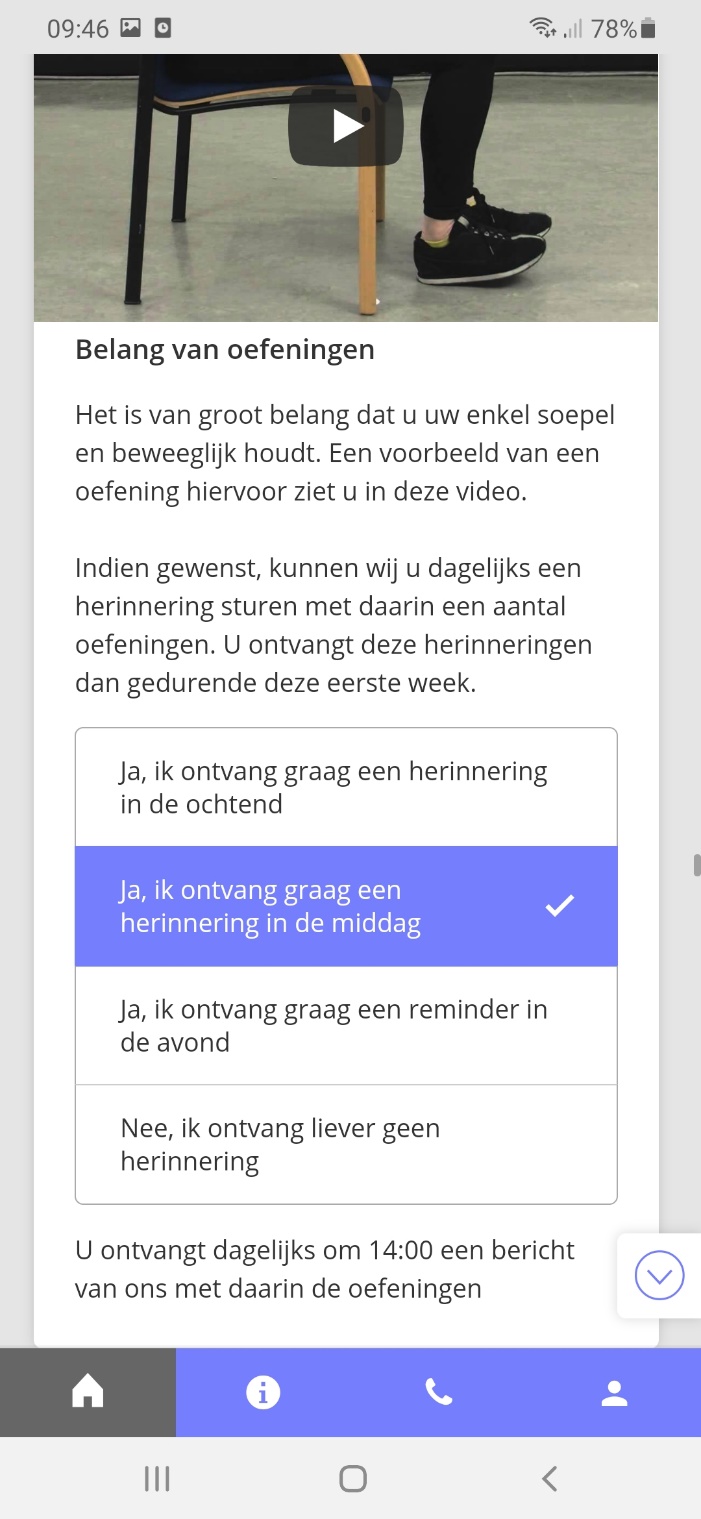


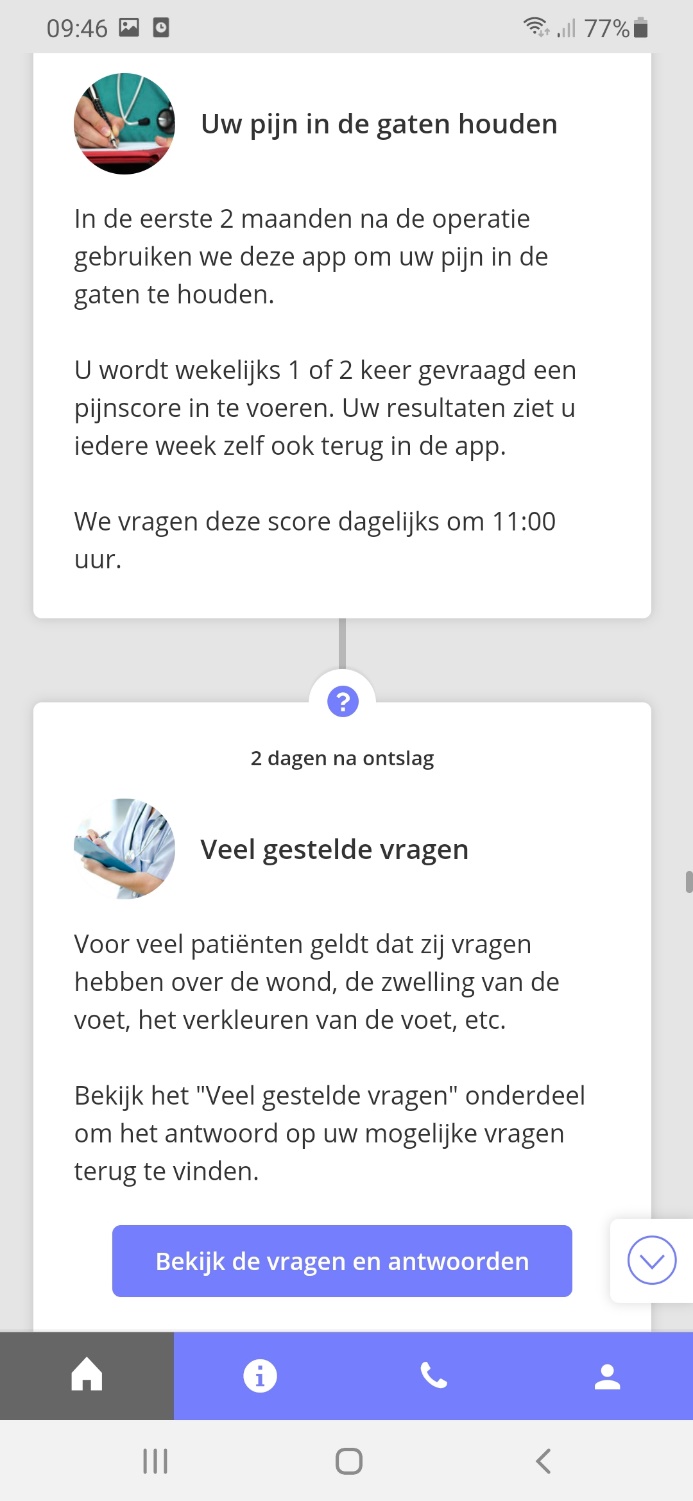

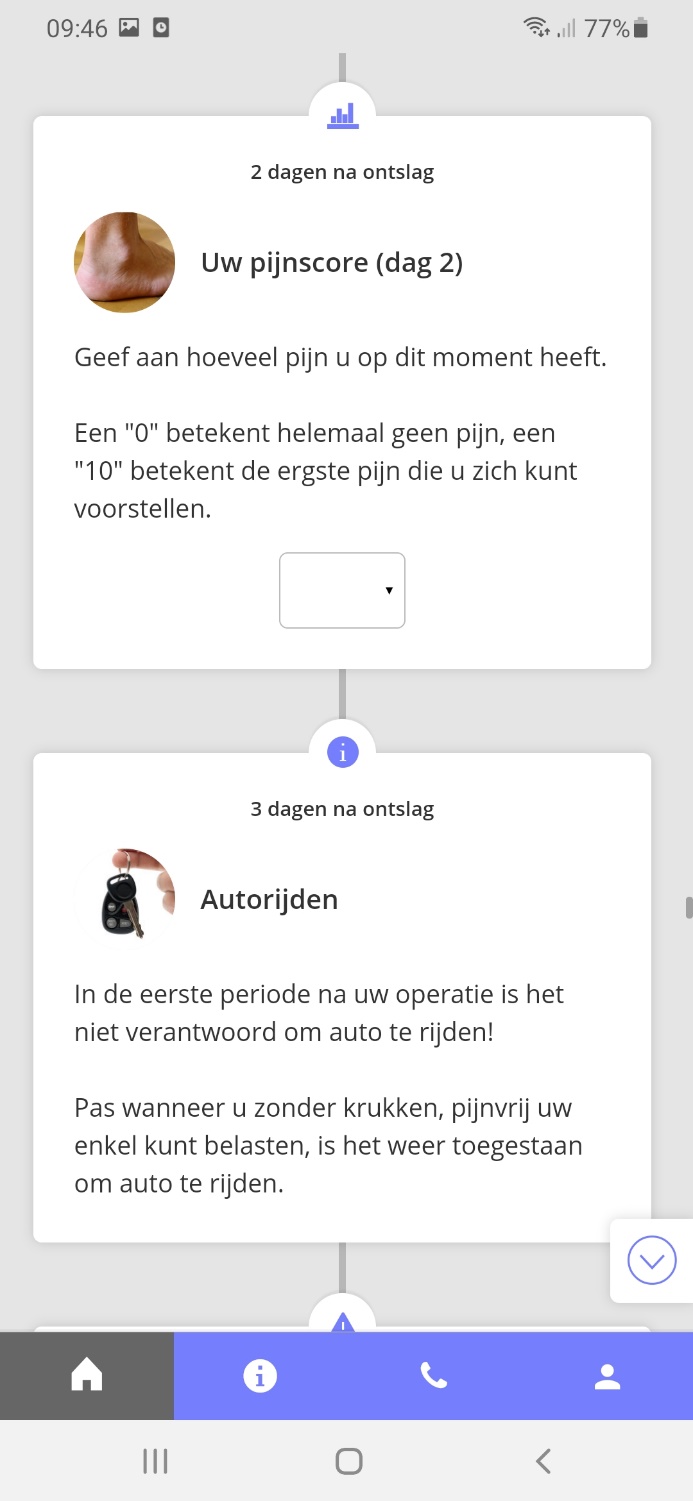


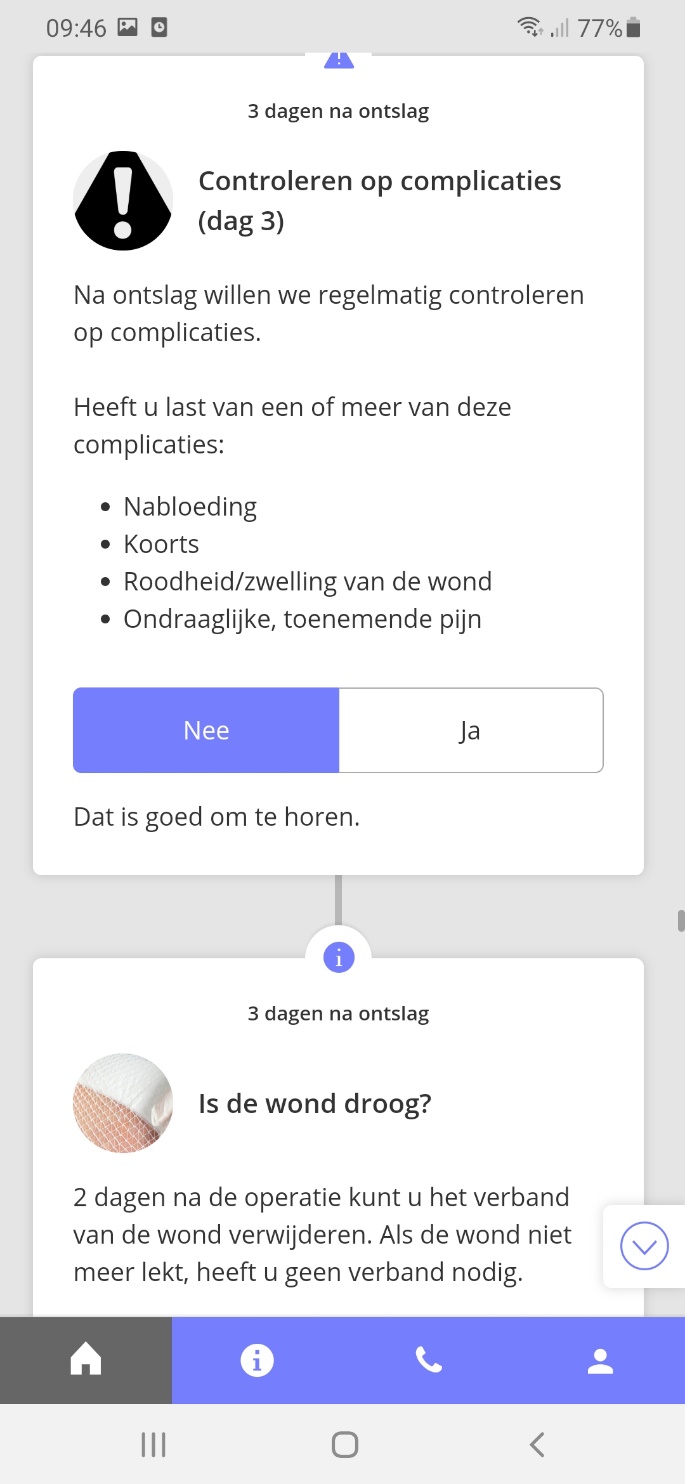

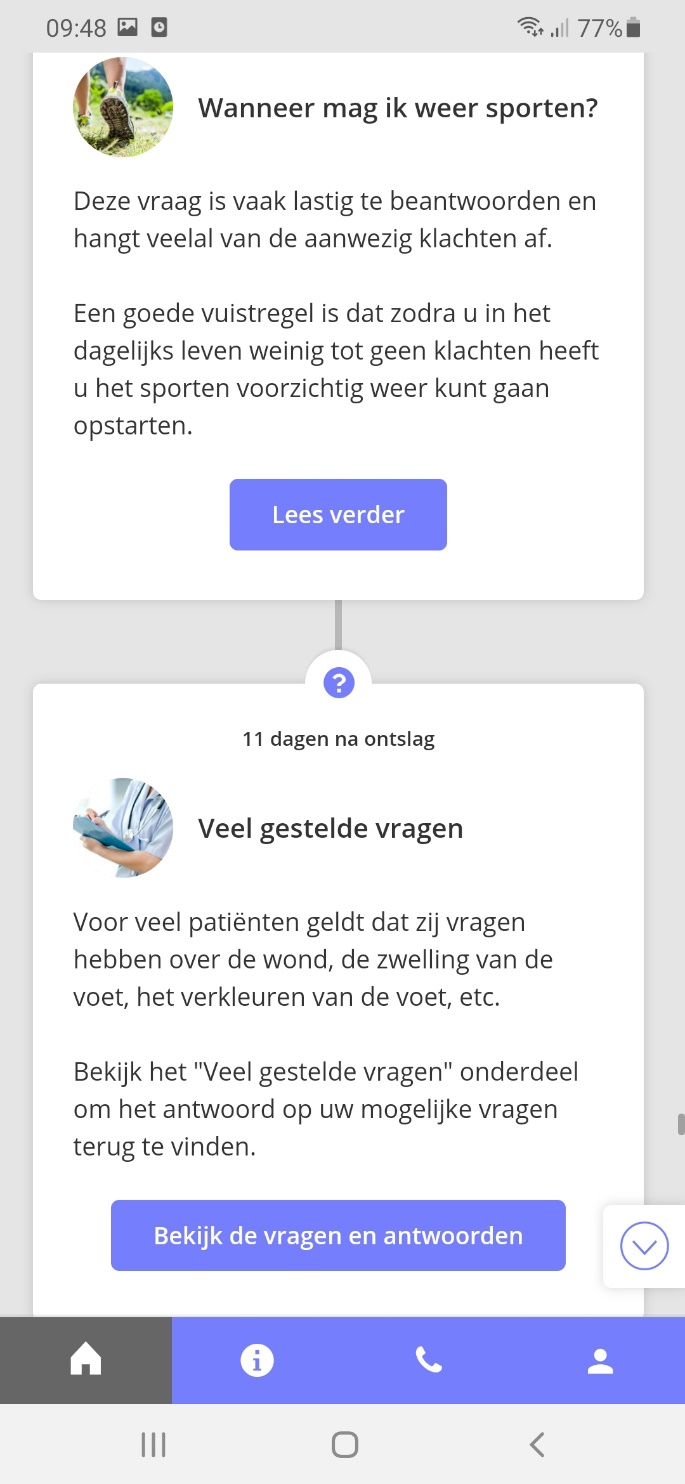


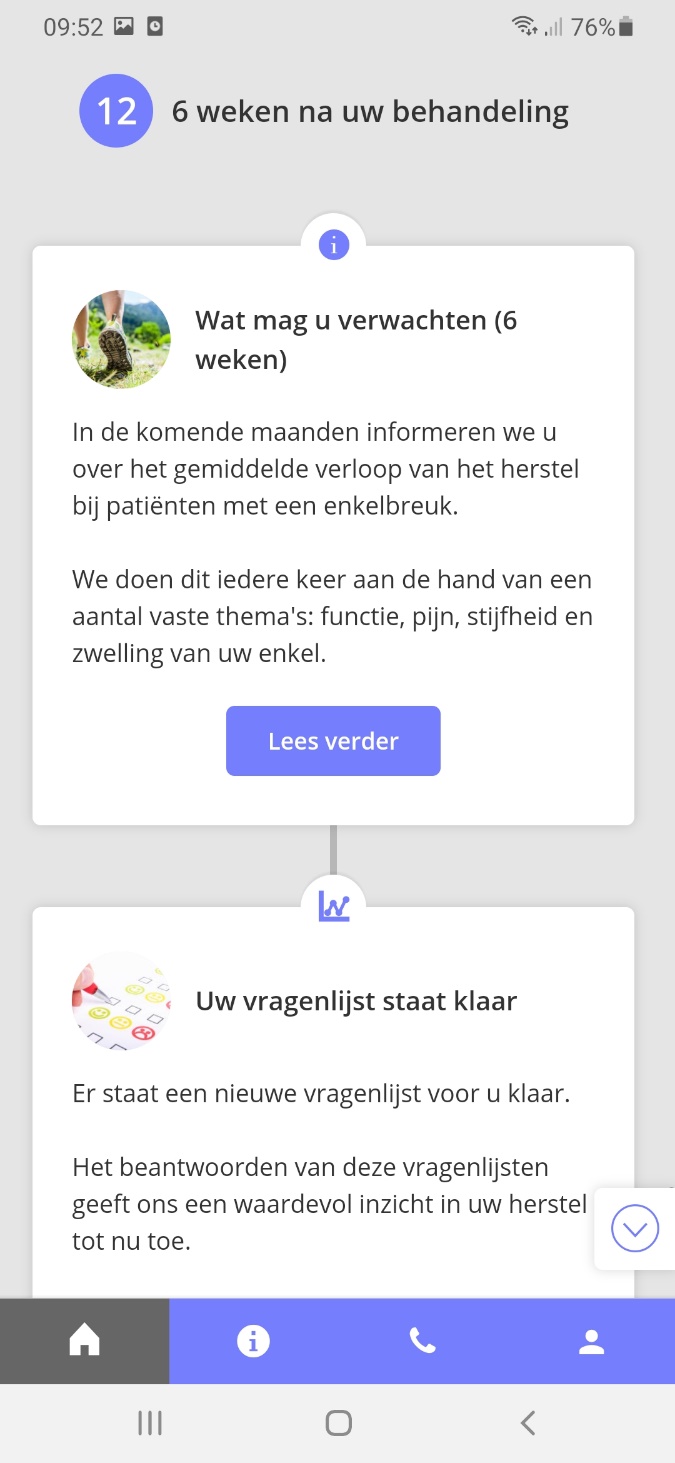

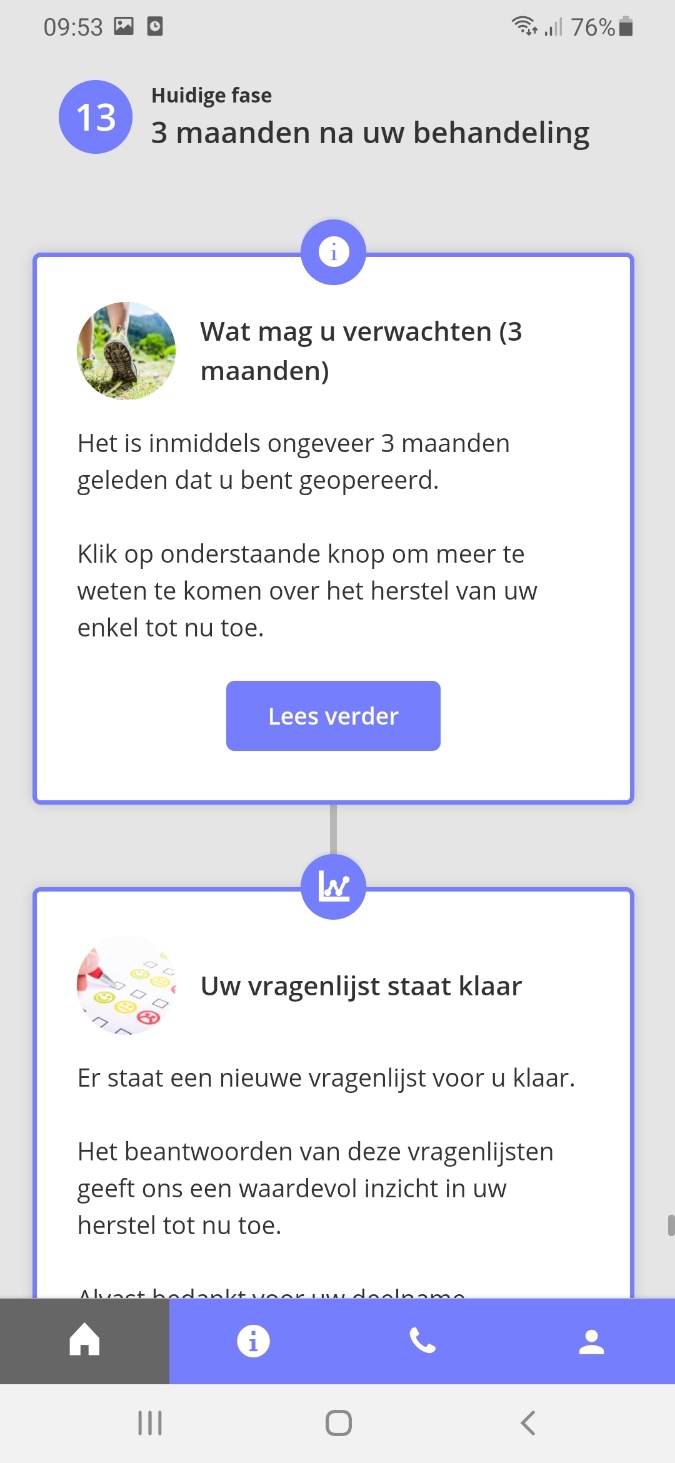

Supplement: Multimedia Appendix 1 [file humanfactors_v9i2e35342_app1.docx]
