# Supplementary material for: Developing mHealth to the Context and Valuation of Injured Patients and Professionals in Hospital Trauma Care: Qualitative and Quantitative Formative Evaluations
Source: JMIR Hum Factors. 2022 Jun 20;9(2):e35342. doi: 10.2196/35342 (PMC9254041; doi:10.2196/35342)
Supplement: Multimedia Appendix 2 [file humanfactors_v9i2e35342_app2.docx]

***Multimedia Appendix 2.*** *Additional details of methods of the discrete choice experiment.*

Part 1: methodological steps

Step 1:

One core team member (MV) made an initial proposal for application attributes and levels based on:

1. A concept of the survey containing a categorization of the attributes that required ‘prioritization’ according to the eHealth workgroup of the Dutch Association of Trauma Surgeons.
2. Idea’s for technology attributes for patient recovery support obtained by exchanging knowledge and sources amongst core team members in preparation of the first round of qualitative evaluation (summarized in the topic list enclosed in Multimedia appendix 1).

Subsequently, other core team members (MdJ, MJ) contributed to refining attribute descriptions and levels. Six attributes were established, with:

- 4 pricing levels
- 3 levels of medical record integration
- 3 levels of local hospital identification and information
- 2 levels of tailoring content to patient level differences through artificial intelligence
- 2 levels of communication tools for complication (e.g. sending a wound picture)
- 2 levels of health outcome monitoring (e.g. patient reported outcome measurement)

Finally, attributes and levels were refined and finalized by collaboration of the researcher (MV) with the workgroup representative (SB).

Step 2:

The following figure is an example of one choice task from the instrument design (original: in Dutch)


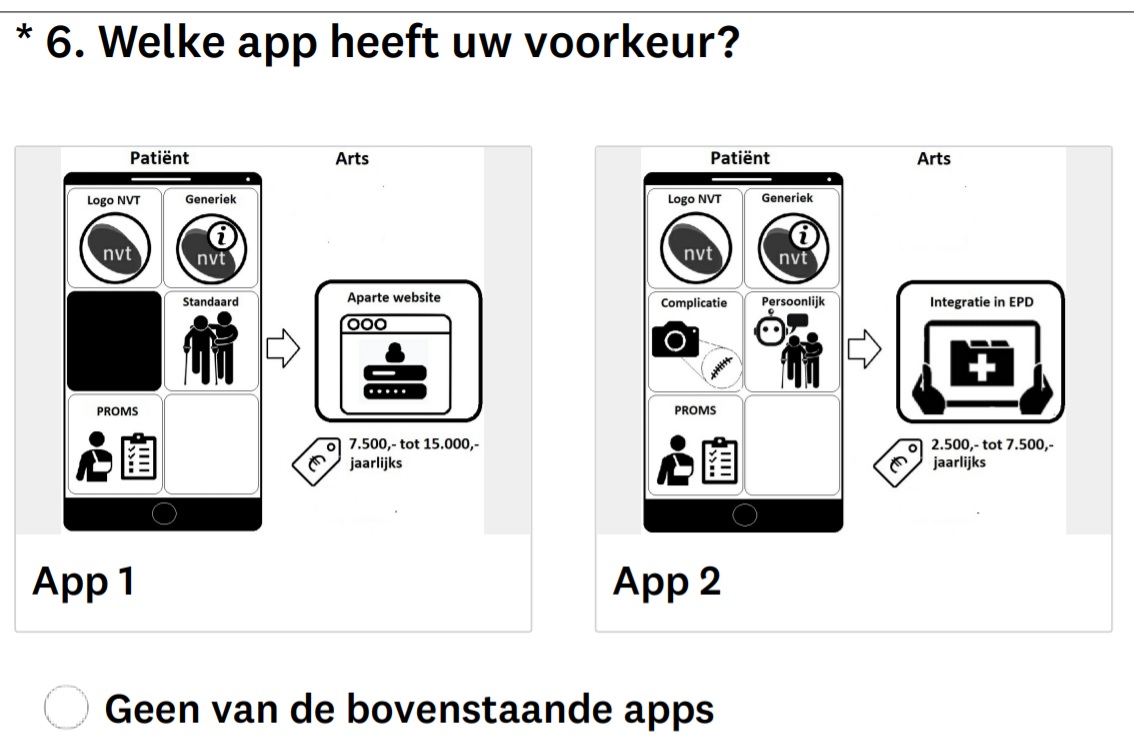


Step 3:

The experimental design was created with the ‘AlgDesign’ package in R. With the ‘gen.factorial’ function in ‘AlgDesign’, a full factorial design was created for 6 attributes with 4, 3, 3, 2, 2, and 2 levels. Next, an optimal fractional design of 11 alternatives was created with the ‘OptFederov’ function (i.e. for D-efficiency optimization). Finally, the choice sets were established as the pairs with same row numbers from the original fractional design and the copy after each was independently sorted in random order. The final design was obtained after repeated runs with different random seeds until all 11 pairs consisted of 2 different alternative attribute level combinations.

Step 4:

Reasons for the survey and the DCE within it by which participants were encouraged to perform the decision tasks were formulated as: "The NVT (Dutch Association of Trauma Surgeons) intends to launch a patient information app ... There are various options to an app, which of course also include costs and time investments ... (the discrete choice experiment is conducted) to get a clear picture of the prioritization of possible attributes of a patient information app and what it may cost for hospitals"

Step 5:

Instrument design is illustrated also by the figure shown above to illustrate the choice tasks.

Step 6:

Data available from the survey regarding participant characteristics, included: years of experience in medical practice, the level of the trauma care center in which they worked, expressed need for collaborative development of a structured patient information application, and rating of importance of reducing patient visits as a goal for developing a patient information application.

Step 7:

Both conditional and mixed logistic regression models were applied for estimating relative preference weights, potential interactions with decision maker groups, and willingness to pay. In all models, attribute level variables were dummy coded, e.g. the three-level attributes are coded with two dummy variables. With each modeling approach, we first estimated prediction weights to inform about the relative importance of the proposed attribute levels (‘model 1’). Model 1 was also the starting point for explorative willingness to pay calculations as ratios between the weights for attribute levels and price. Further exploration involved the additions of several theoretically or contextually plausible interaction effects between attribute levels and decision maker characteristics (‘model 2’). Model fit was evaluated with likelihood ratio chi-square tests, McFadden’s pseudo R-squared, and the Bayesian Information Criterion (BIC).

*Conditional logit*

Conditional logistic regression has the advantages – to more sophisticated approaches of statistically modeling DCE data - of being the most familiar (mostly used), resulting in a single optimal solution, and being less data intensive. However, predictions can be biased at violation of the assumption that attribute level preference weights are of a similar size and relative strength across decision makers. For example, it is not accounted for that certain decision makers may prefer certain combinations of attribute levels, or that certain decision makers values attributes outside the model relatively more.

The first conditional logit model specifies preference estimates for the level attributes. The systemic part of the decision prediction model (U = utility) is the linear combination of:

U = β_0_* Alternative specific constant (ASC) +

β_1_*A1 Own label display + β_2_*A1: local information adjustment +

β_3_*A2 Complication detection +

β_4_*’A3 Personalized rehabilitation plans through artificial intelligence +

β_5_*A4 Patient Reported Outcome Measures +

β_6_*A5 Electronic Medical Record (EMR) integration + β_7_*A5: Web-viewer +

β_8_*A6 price level 1 + β_9_*A6: price level 2 + β_10_*A6: price level 3

Herein, the weight for the ASC quantifies probability of choosing an application, rather than opting out, when all attributes are at their lowest levels (all dummies are ‘0’). β1 and β2 are the preference weights for two dummies that represent the three-level attribute of hospital identification and information adjustment against the reference level (both dummies are ‘0’) of generic labels and information. The preference weight for including communication possibilities for complication detection, like sending in wound pictures, is expressed by β_3._ The preference weight for advanced artificial intelligence for personalization versus a standardized ‘one-size-fits-all’ rehabilitation plan is β_4._ Preference for including patient health monitoring like patient reported outcome measures is weighted by β_5._  Preference weights for different levels of convenient access to patient data collected by means of the app, including full Electronic Medical Record integration, a pragmatic solution (web-viewer), or logging in on an external website (reference) are given β_6_ and β_7._ Finally, β_8_ to β_10_ are weights for different yearly price levels. The reference (4^th^) level represents usage free of charge.

In a second model, decision maker characteristics are added to the linear combination in the form of fixed interaction effects. For example, by adding:

‘… + β_11_* ASC: less than 10 years of experience in trauma surgery

Herein, ‘:’ follows the notation of interaction in the ‘gmnl’ formula. Each interaction term added required a plausible rationale: theoretically, contextually, or empirically. Regarding the example given, several previous studies showed that medical professionals who are earlier in their careers have more incentive to invest effort in adopting novel eHealth tools(1). Therefore, professionals who are at an earlier stage in their careers may be less likely to opt out. The participant characteristics were coded as follows:

1. Years of experience in medical practice was coded as; 0-10 years = 1 & other (more) = 0.
2. (Lowest) level of the trauma care center in which they worked was coded as level 1 = 1 & other = 0.
3. Expressed need for collaborative development of a structured patient information application was coded as; (very) much = 1 & other = 0.
4. Rating of importance of reducing patient visits as a goal of the application was coded as; (very) important = 1 & other = 0.

In addition to these fixed elements, responses depended on the panel structure of DCE data and random measurement error, which is assumed to follow an independent and identical distributed type 1 extreme-value distribution.

*Mixed logit*

To take potential heterogeneity of ‘scale’ and ‘preference’ into account, mixed logit models allow preference weights to randomly vary across subjects and correlate amongst each other. In this case, we allowed for the random variation of the preference weights, but not for correlations amongst the preference weights because the number of parameters to be estimated may become problematically large relative to the number of participants in our convenience sample. Accordingly, the mixed logit model included estimates for the means as well as the standard deviations of the weights for the ASC and attribute levels. Compared to the conditional logit models specifications (model 1 and model 2), mixed logit models were specified - with the gmnl function – by:

- The same formulas of attribute levels and interactions with participant characteristics
- Assigning each attribute level as random, normally distributed, parameters.
- Model type was set to ‘mixl’

Furthermore, the sensitivity of the estimations was checked to the number of draws of pseudo random numbers for parameter estimation (i.e.: 500, 1000, 2000, and 5000). Decisions regarding which results to report were made based on the model fit indices. Finally, a model based on 2000 draws was selected.

Part 2: Detailed results

The following table describes the full results of the conditional logit models

|  | **Model 1** | | | **Model 2** | | |
| --- | --- | --- | --- | --- | --- | --- |
| **Attribute levels** | b(se) | Exp(b) | *P* | b(se) | Exp(b) | *P* |
| Alternative specific constant (ASC) | -1.46 (.19) | 0.23 | <.001 | -2.36 (.22) | 0.09 | <.001 |
| Own label display | 0.51 (.12) | 1.66 | <.001 | 0.52 (.12) | 1.68 | <.001 |
| Local content adjustment | 0.60 (.13) | 1.82 | <.001 | 0.61 (.13) | 1.84 | <.001 |
| Complication detection | 0.40 (.11) | 1.49 | <.001 | 0.23 (.13) | 1.26 | .08 |
| Artificial intelligence | 0.31 (.09) | 1.36 | .001 | 0.31 (.09) | 1.36 | <.001 |
| Patient Reported Outcome Measures | 0.70 (.10) | 2.01 | <.001 | 0.70 (.11) | 2.02 | <.001 |
| Electronic Medical Record integration | 1.20 (.14) | 3.33 | <.001 | 1.21 (.14) | 3.56 | <.001 |
| Web-viewer in EMR | 0.85 (.18) | 2.33 | <.001 | 0.82 (.18) | 2.28 | <.001 |
| Price < €2.500 | -0.20 (.18) | 0.82 | .26 | -0.22 (.19) | 0.80 | .23 |
| Price €2.500 - €7.500 | -0.41 (.16) | 0.66 | .01 | -.42 (.16) | 0.66 | .01 |
| Price €7.500 - €15.000 | -0.47 (.15) | 0.62 | .002 | -.48 (.15) | 0.62 | .002 |
| ASC: collective development need |  |  |  | 0.79 (.14) | 2.19 | <.001 |
| ASC: not more than 10 years of experience in medical practice |  |  |  | 0.78 (.14) | 2.20 | <.001 |
| Complication detection: rated importance of reducing clinical visits |  |  |  | 0.31 (.14) | 1.36 | .03 |
|  |  |  |  |  |  |  |
| **Model fit indices** | Fit index | df | *P* | Fit index | df | *P* |
| Likelihood Chi-square | 218.6 | 11 | <.001 | 292.83 | 14 | <.001 |
| Bayesian information criterion | 2493.8 |  |  | 2428,5 |  |  |
| McFadden’s pseudo R-squared | 0.08 |  |  | 0.11 |  |  |

The next table describes the full results of the mixed logit models.

|  | **Model 1** | | | | | | **Model 2** | | | | | |  |
| --- | --- | --- | --- | --- | --- | --- | --- | --- | --- | --- | --- | --- | --- |
| **Estimates of attribute levels** | b(se) | | Exp(b) | | *P* | | b(se) | | Exp(b) | | *P* | |  |
| Alternative specific constant | -1.50 (.40) | | .22 | | <.001 | | -3.85 (.87) | | 0.02 | | <.001 | |  |
| Own label display | 0.75 (.26) | | 2.11 | | .004 | | 0.71 (.25) | | 2.04 | | .005 | |  |
| Local content adjustment | 1.09 (.26) | | 2.99 | | <.001 | | 1.04 (.25) | | 2.84 | | <.001 | |  |
| Complication detection | 0.79 (.23) | | 2.22 | | <.001 | | 0.41 (.29) | | 1.51 | | .15 | |  |
| Artificial intelligence | 0.54 (.19) | | 1.71 | | .006 | | 0.52 (.18) | | 1.67 | | .004 | |  |
| PROMS | 1.38 (.24) | | 3.98 | | <.001 | | 1.28 (.23) | | 3.60 | | <.001 | |  |
| EMR integration | 2.14 (.31) | | 8.49 | | <.001 | | 2.13 (.30) | | 8.39 | | <.001 | |  |
| Web-viewer in EMR | 1.24 (.32) | | 3.71 | | <.001 | | 1.11 (.31) | | 3.04 | | <.001 | |  |
| Price < €2.500 | -0.62 (.34) | | 0.54 | | .07 | | -0.52 (.33) | | 0.59 | | .11 | |  |
| Price €2.500 - €7.500 | -0.93 (.30) | | 0.40 | | .002 | | -.87 (.29) | | 0.42 | | .003 | |  |
| Price < €7.500 - €15.000 | -0.88 (.25) | | 0.42 | | <.001 | | -.80 (.24) | | 0.45 | | .001 | |  |
| ASC: collective development need |  | |  | |  | | 2.03 (.81) | | 7.63 | | .01 | |  |
| ASC: not more than 10 years of experience in medical practice |  | |  | |  | | 1.42 (.66) | | 4.13 | | .03 | |  |
| Complication detection: rated importance of reducing clinical visits |  | |  | |  | | 0.47 (.36) | | 1.60 | | .20 | |  |
|  |  | |  | |  | |  | |  | |  | |  |
| **Standard deviations** | **SD** | |  | |  | |  | |  | |  | |  |
| Alternative specific constant | 3.44 (.49) | |  | | <.001 | | 3.29 (.44) | |  | | <.001 | |  |
| Own label display | 1.96 (.28) | |  | | <.001 | | 1.75 (.26) | |  | | <.001 | |  |
| Local content adjustment | 1.63 (.32) | |  | | <.001 | | 1.32 (.29) | |  | | <.001 | |  |
| Complication detection | 1.41 (.24) | |  | | <.001 | | 1.24 (.22) | |  | | <.001 | |  |
| Artificial intelligence | 1.19 (.22) | |  | | <.001 | | 1.02 (.23) | |  | | <.001 | |  |
| PROMS | 1.27 (.31) | |  | | <.001 | | 1.13 (.25) | |  | | <.001 | |  |
| EMR integration | 2.10 (.33) | |  | | <.001 | | 1.67 (.32) | |  | | <.001 | |  |
| Web-viewer in EMR | 1.47 (.39) | |  | | <.001 | | 1.26 (.46) | |  | | .006 | |  |
| Price < €2.500 | 0.99 (.52) | |  | | .06 | | 0.95 (.44) | |  | | .03 | |  |
| Price €2.500 - €7.500 | 1.19 (.45) | |  | | .008 | | 1.21 (.35) | |  | | .001 | |  |
| Price €7.500 - €15.000 | 0.95 (.32) | |  | | .003 | | 0.84 (.40) | |  | | .04 | |  |
|  |  | |  | |  | |  | |  | |  | |  |
| **Model fit indices** | | | Fit index | | df | | *P* | | Fit index | | df | | *P* |
| Likelihood Chi-square | 710.2 | | 22 | | <.001 | |  | |  | |  | |  |
| Bayesian information criterion | 2067.9 | |  | |  | |  | |  | |  | |  |
| McFadden’s pseudo R-squared | 0.27 | |  | |  | |  | |  | |  | |  |

Finally, willingness to pay data, corresponding to the figure 5 in the paper are available upon request.

Reference:

Leigh S, Ashall-Payne L, Andrews T. Barriers and Facilitators to the Adoption of Mobile Health Among Health Care Professionals From the United Kingdom: Discrete Choice Experiment. JMIR Mhealth Uhealth. 2020;8(7):e17704.
